# Supplementary material for: Layer Analysis Based on RNA-Seq Reveals Molecular Complexity of Gastric Cancer
Source: Int J Mol Sci. 2024 Oct 22;25(21):11371. doi: 10.3390/ijms252111371 (PMC11545517; doi:10.3390/ijms252111371)
Supplement: Supplementary file 1 [file ijms-25-11371-s001.zip › ijms-3229845-supplementary.pdf]

Supplementary Figure S1: Flowchart of inclusion criteria of gastric adenocarcinoma patients. GA: Gastric adenocarcinoma. 5-FU: 5-fluorouracil. TCGA: The Cancer Genome Atlas.

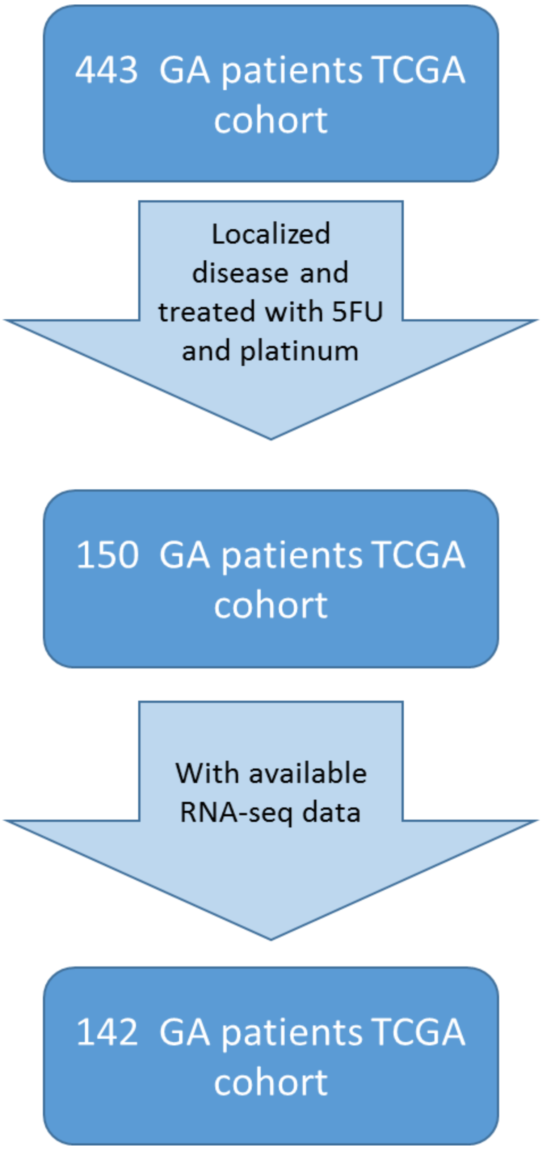

Supplementary Figure S2. Overall survival based on TCGA subgroups classification after categorizing the non-assigned (NA) tumors.

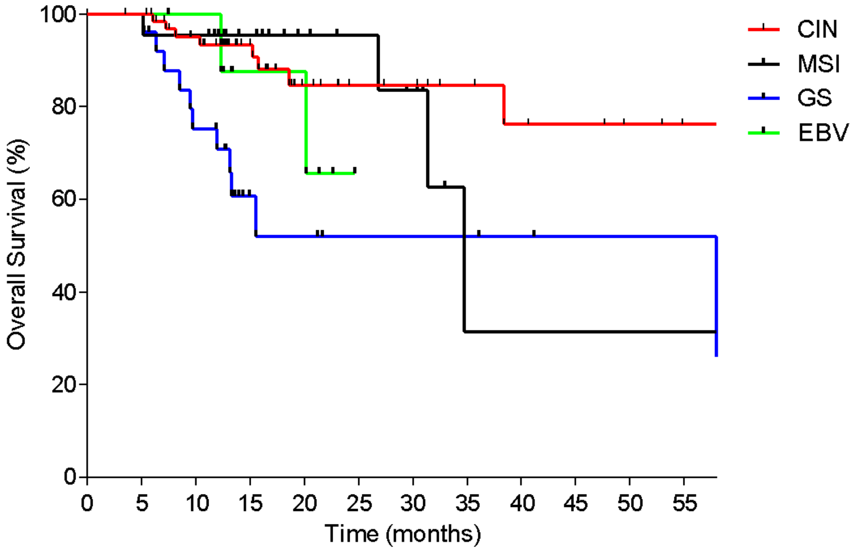

P value	0.0079

Supplementary Figure S3. Functional node activity of TCGA gastric adenocarcinoma molecular subtype groups. CIN: Chromosome instability. MSI: Microsatellite instability. GS: Genomically stable. EBV: Epstein-Barr virus.

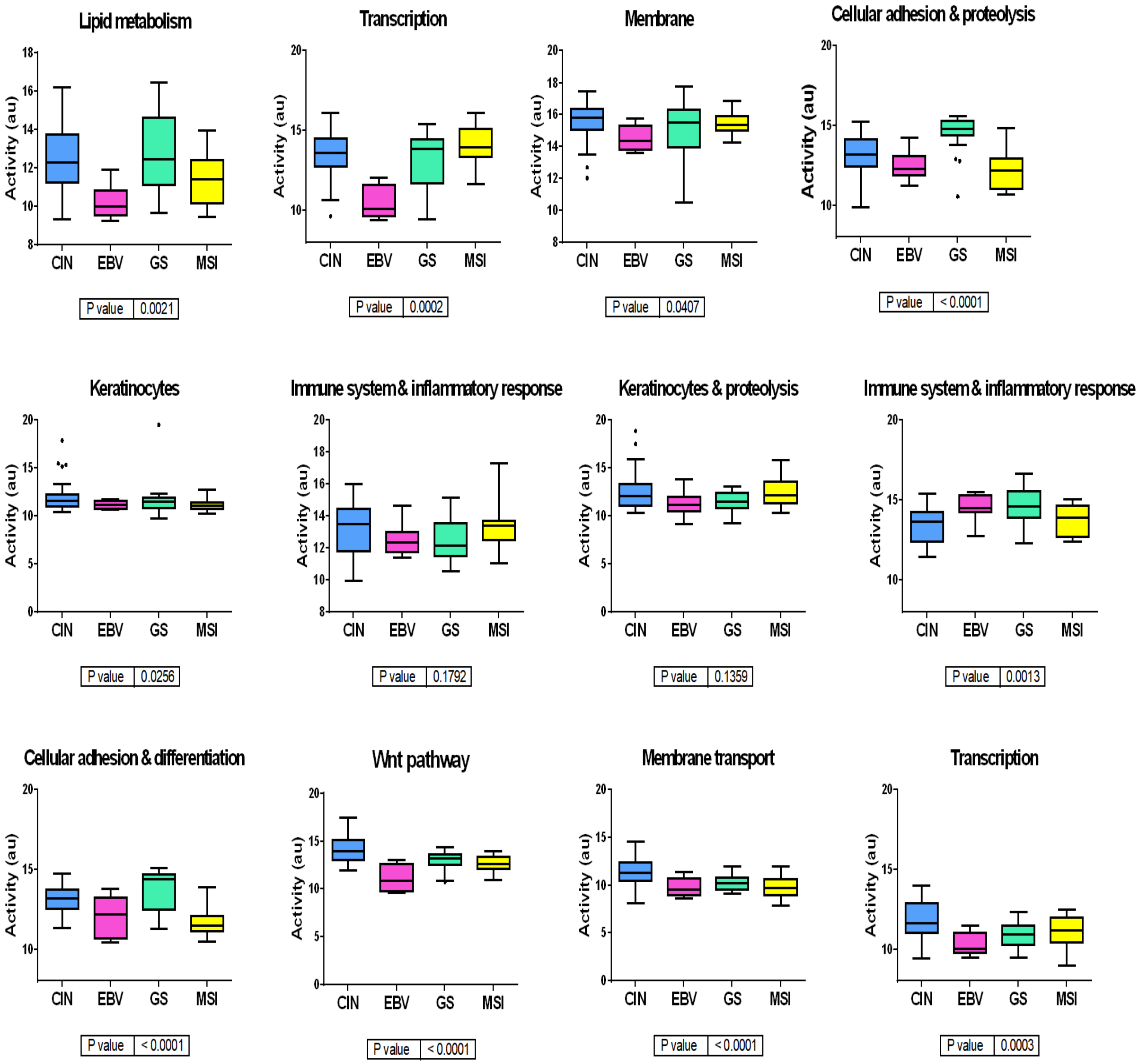

Supplementary Figure S4: TCGA subtypes and clusters defined by each layer

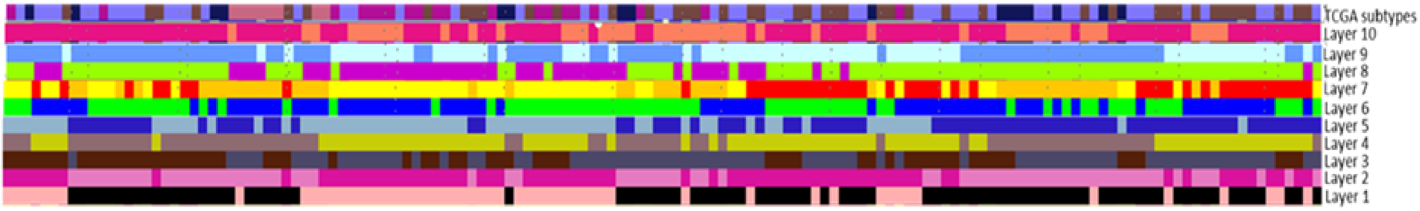

Supplementary Figure S5. (A) Heatmap network of combined molecular layer (CML) groups **(B)** Comparison of functional node activities between defined CML groups. ****, $p < 0.0001$; ***, $p < 0.001$; **, $p < 0.01$; *, $p < 0.05$

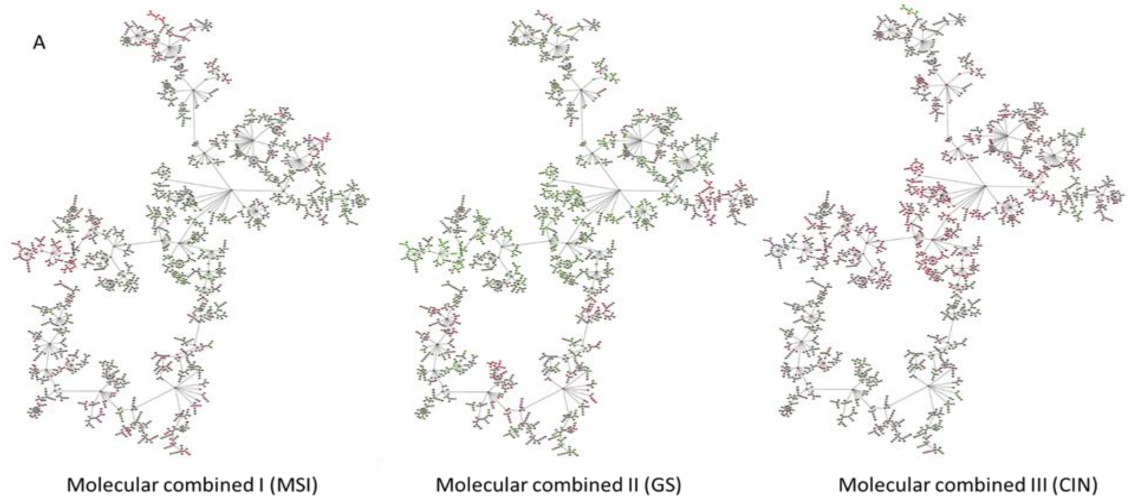

B

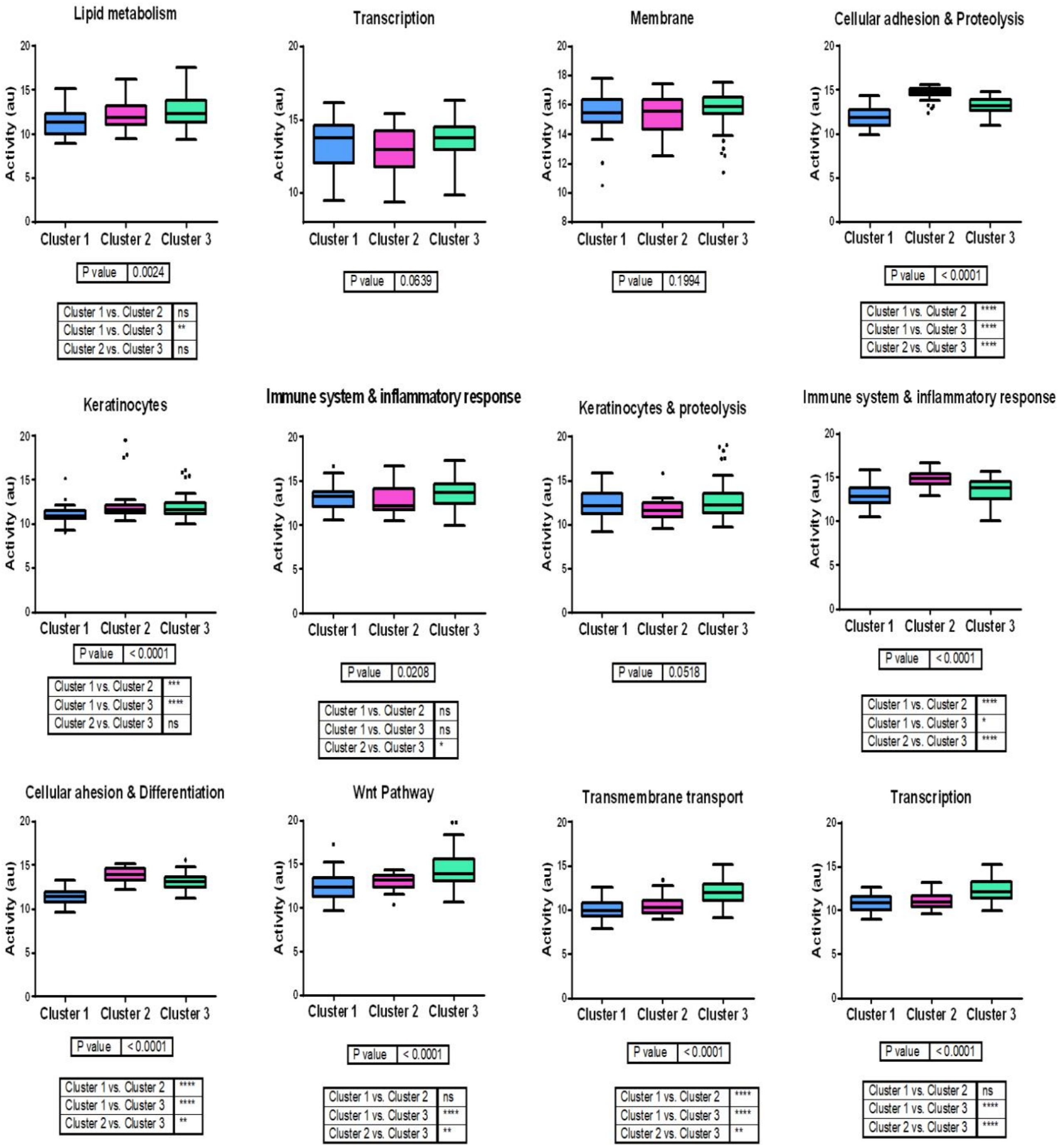
Lipid metabolism
Activity (au)
Cluster 1 Cluster 2 Cluster 3
P value 0.0024
Cluster 1 vs. Cluster 2 ns
Cluster 1 vs. Cluster 3 **
Cluster 2 vs. Cluster 3 ns
Transcription
Activity (au)
Cluster 1 Cluster 2 Cluster 3
P value 0.0639
Membrane
Activity (au)
Cluster 1 Cluster 2 Cluster 3
P value 0.1994
Cellular adhesion & Proteolysis
Activity (au)
Cluster 1 Cluster 2 Cluster 3
P value < 0.0001
Cluster 1 vs. Cluster 2 ****
Cluster 1 vs. Cluster 3 ****
Cluster 2 vs. Cluster 3 ****
Keratinocytes
Activity (au)
Cluster 1 Cluster 2 Cluster 3
P value < 0.0001
Cluster 1 vs. Cluster 2 ***
Cluster 1 vs. Cluster 3 ****
Cluster 2 vs. Cluster 3 ns
Immune system & inflammatory response
Activity (au)
Cluster 1 Cluster 2 Cluster 3
P value 0.0208
Cluster 1 vs. Cluster 2 ns
Cluster 1 vs. Cluster 3 ns
Cluster 2 vs. Cluster 3 *
Keratinocytes & proteolysis
Activity (au)
Cluster 1 Cluster 2 Cluster 3
P value 0.0518
Immune system & inflammatory response
Activity (au)
Cluster 1 Cluster 2 Cluster 3
P value < 0.0001
Cluster 1 vs. Cluster 2 ****
Cluster 1 vs. Cluster 3 *
Cluster 2 vs. Cluster 3 ****
Cellular ahesion & Differentiation
Activity (au)
Cluster 1 Cluster 2 Cluster 3
P value < 0.0001
Cluster 1 vs. Cluster 2 ****
Cluster 1 vs. Cluster 3 ****
Cluster 2 vs. Cluster 3 **
Wnt Pathway
Activity (au)
Cluster 1 Cluster 2 Cluster 3
P value < 0.0001
Cluster 1 vs. Cluster 2 ns
Cluster 1 vs. Cluster 3 ****
Cluster 2 vs. Cluster 3 **
Transmembrane transport
Activity (au)
Cluster 1 Cluster 2 Cluster 3
P value < 0.0001
Cluster 1 vs. Cluster 2 ****
Cluster 1 vs. Cluster 3 ****
Cluster 2 vs. Cluster 3 **
Transcription
Activity (au)
Cluster 1 Cluster 2 Cluster 3
P value < 0.0001
Cluster 1 vs. Cluster 2 ns
Cluster 1 vs. Cluster 3 ****
Cluster 2 vs. Cluster 3 ****

Supplementary Figure S6. Molecular combined layer (CML) reflecting differences in functional node activity levels in the CIN TCGA subtype. **(A)** Heatmap network of layer groups, CIN in CML1 (CIN-MSI-like) (A.1) or CML3 (CIN classical) (A.2). **(B)** Comparison of functional node activities between defined groups. ****, $p < 0.0001$; ***, $p < 0.001$; **, $p < 0.01$; *, $p < 0.05$

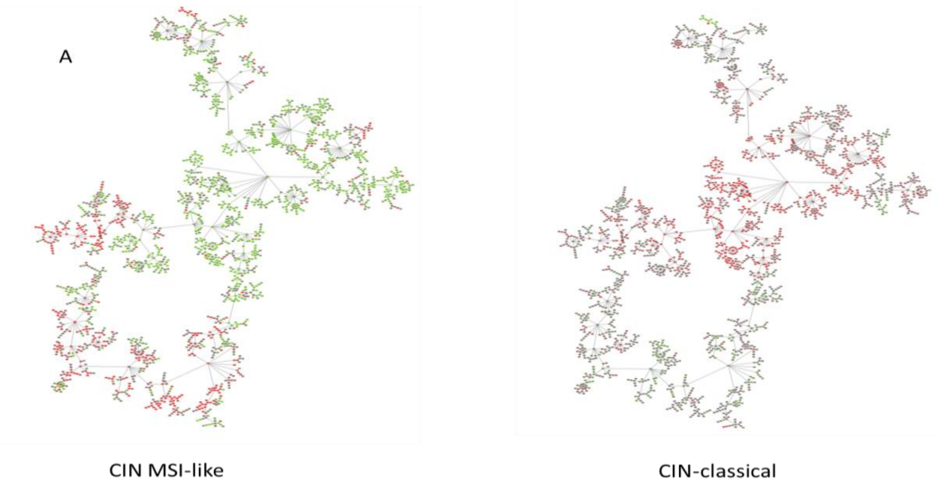

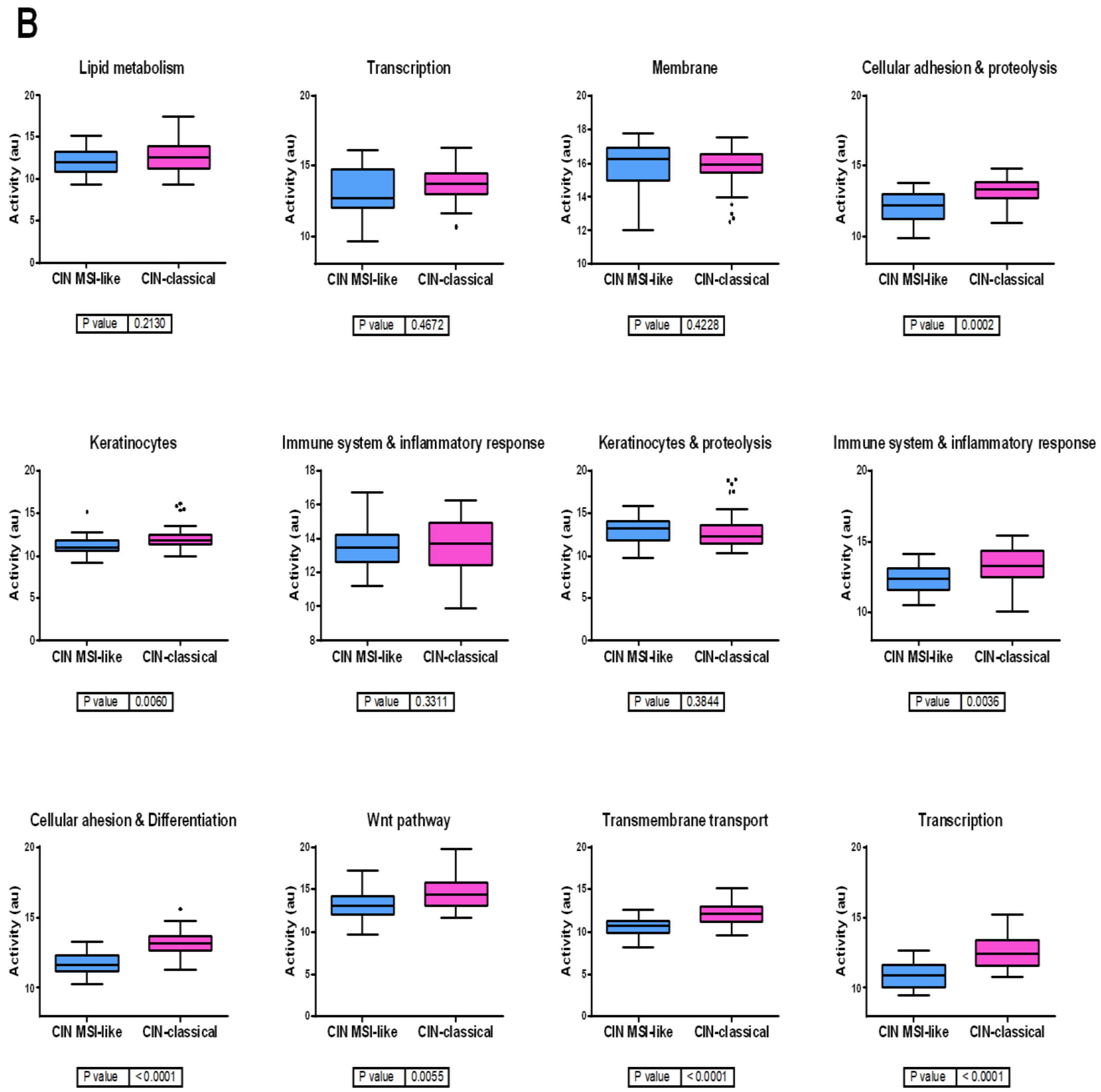

Supplementary Figure S7: Proteolysis layer reflecting differences in functional node activity levels. **(A)** Heatmap network of layer groups. **(B)** Comparison of functional node activities between defined groups. ****, $p < 0.0001$; ***, $p < 0.001$; **, $p < 0.01$; *, $p < 0.05$. **(C)** Overall survival of the two groups defined on the proteolysis layer.

A

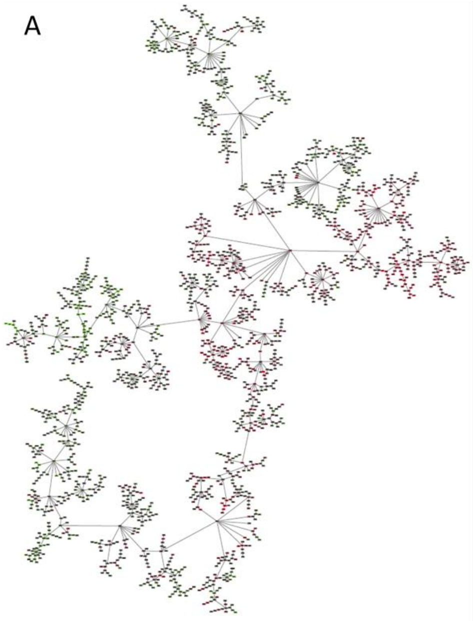

Cluster 1

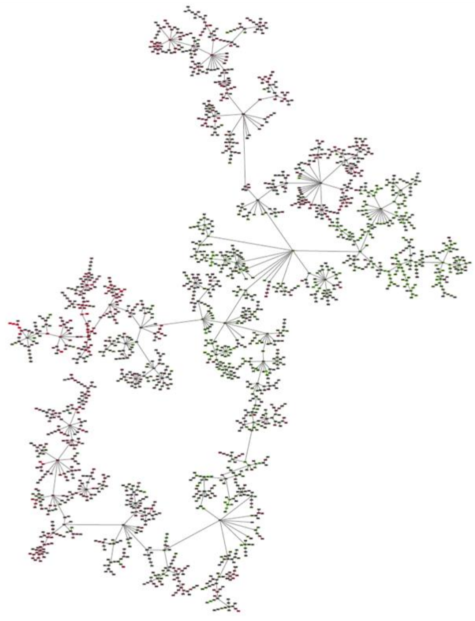

Cluster 2

B

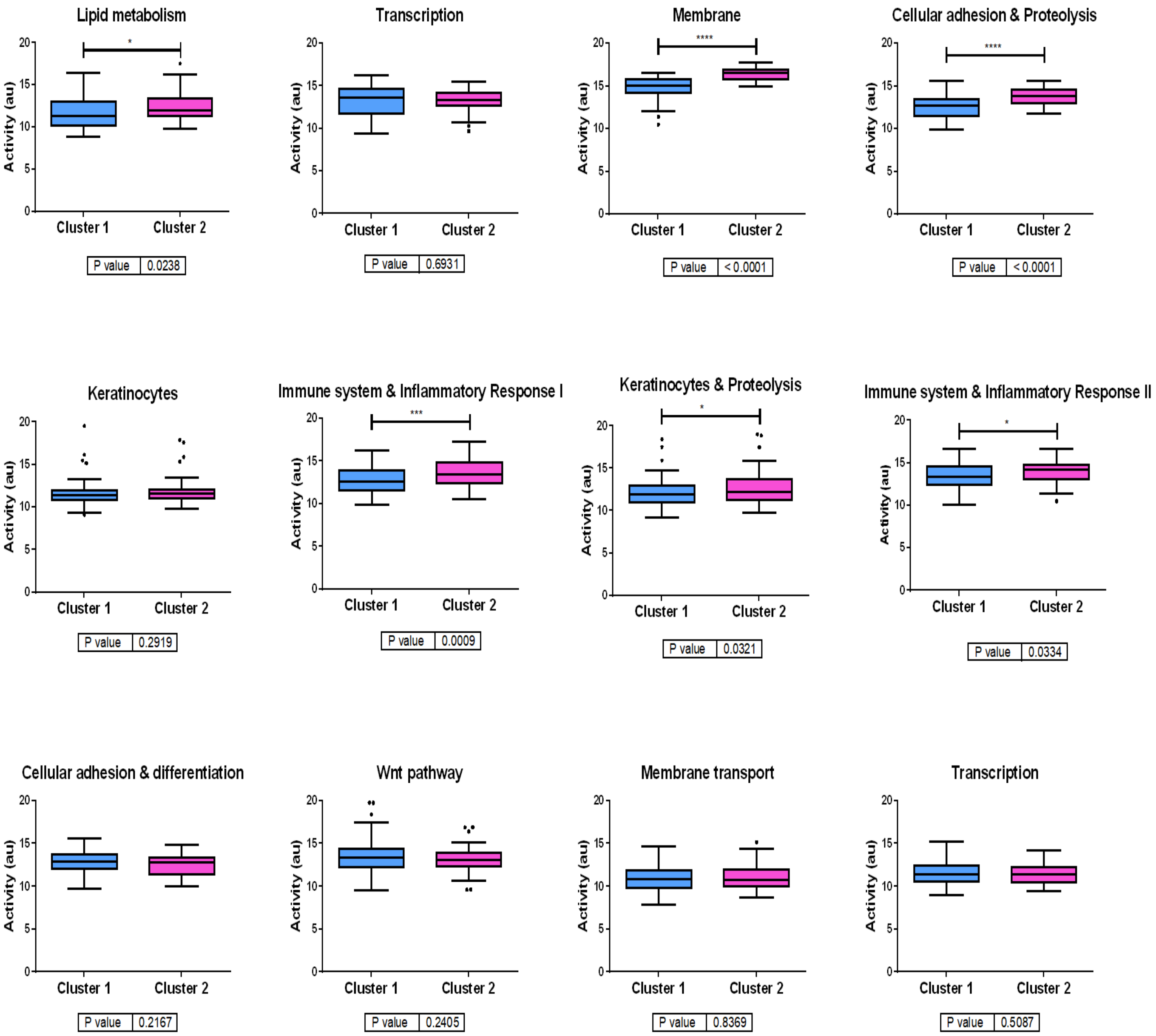
Lipid metabolism
*
Activity (au)
Cluster 1
Cluster 2
P value 0.0238
Transcription
Activity (au)
Cluster 1
Cluster 2
P value 0.6931
Membrane
****
Activity (au)
Cluster 1
Cluster 2
P value < 0.0001
Cellular adhesion & Proteolysis
****
Activity (au)
Cluster 1
Cluster 2
P value < 0.0001
Keratinocytes
Activity (au)
Cluster 1
Cluster 2
P value 0.2919
Immune system & Inflammatory Response I
***
Activity (au)
Cluster 1
Cluster 2
P value 0.0009
Keratinocytes & Proteolysis
*
Activity (au)
Cluster 1
Cluster 2
P value 0.0321
Immune system & Inflammatory Response II
*
Activity (au)
Cluster 1
Cluster 2
P value 0.0334
Cellular adhesion & differentiation
Activity (au)
Cluster 1
Cluster 2
P value 0.2167
Wnt pathway
Activity (au)
Cluster 1
Cluster 2
P value 0.2405
Membrane transport
Activity (au)
Cluster 1
Cluster 2
P value 0.8369
Transcription
Activity (au)
Cluster 1
Cluster 2
P value 0.5087

C)

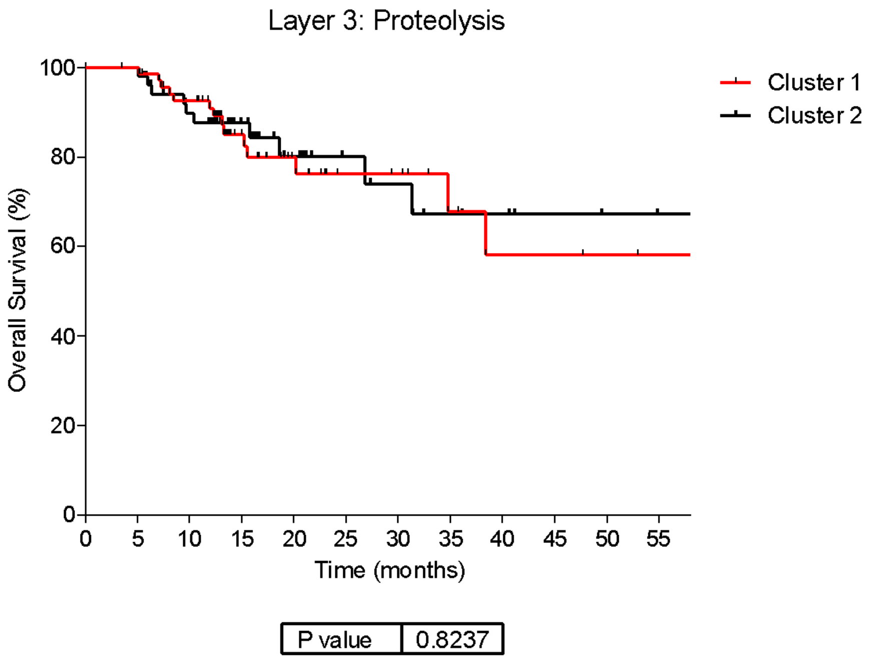

Supplementary Figure S8: Lipid metabolism layer reflecting differences in functional node activity levels. **(A)** Heatmap network of layer groups. **(B)** Comparison of functional node activities between defined groups. ****, p < 0.0001; ***, p < 0.001; **, p < 0.01; *, p < 0.05. **(C)** Overall survival of the two groups defined on the lipid metabolism layer.

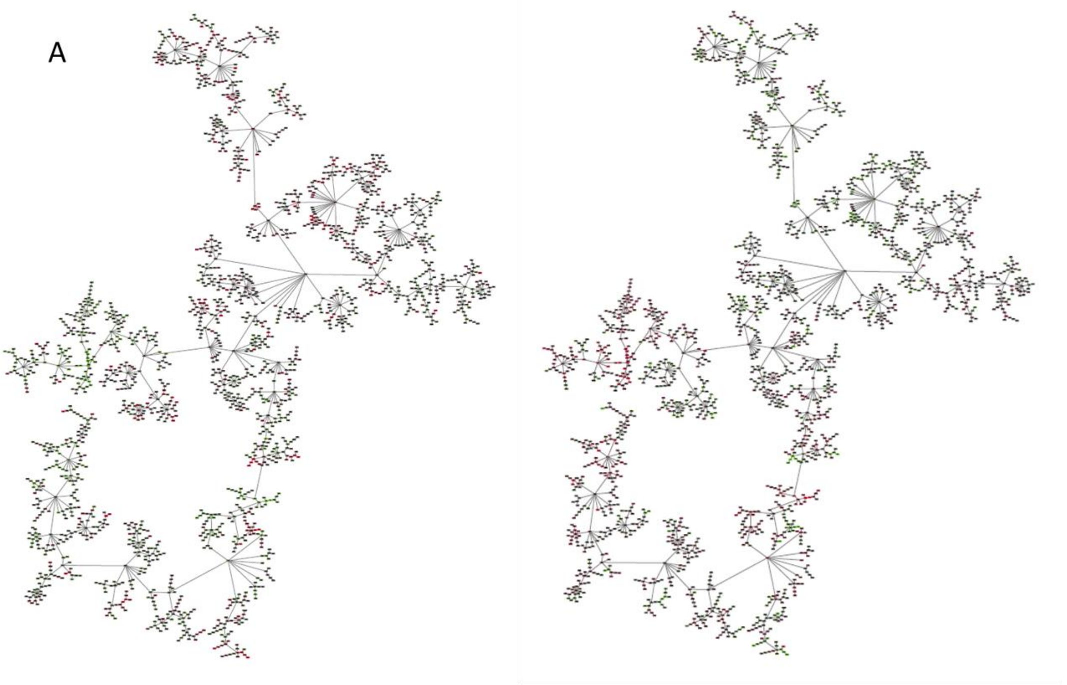

Cluster 1 Cluster 2

B

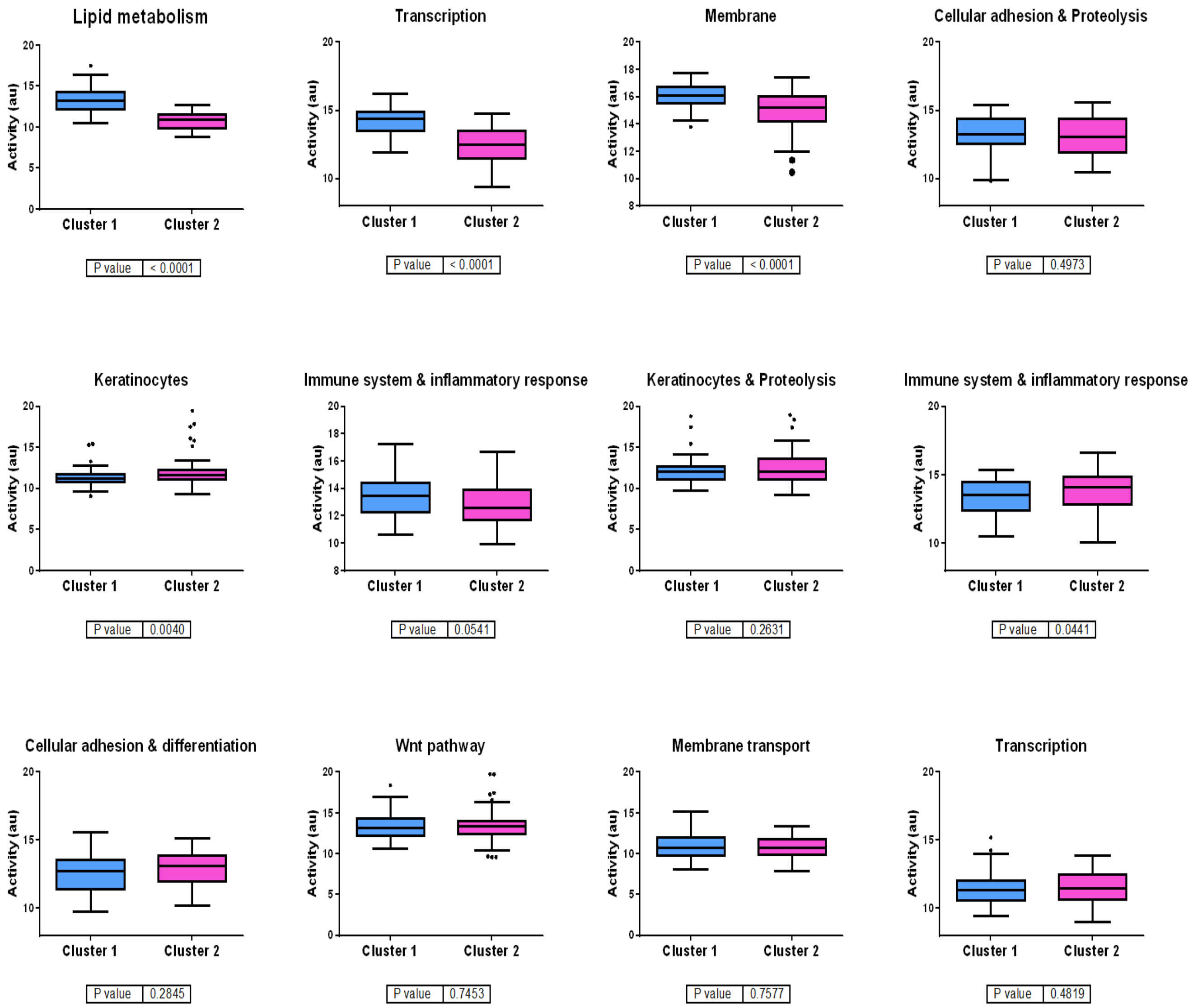
Lipid metabolism
Activity (au)
Cluster 1
Cluster 2
P value | < 0.0001
Transcription
Activity (au)
Cluster 1
Cluster 2
P value | < 0.0001
Membrane
Activity (au)
Cluster 1
Cluster 2
P value | < 0.0001
Cellular adhesion & Proteolysis
Activity (au)
Cluster 1
Cluster 2
P value | 0.4973
Keratinocytes
Activity (au)
Cluster 1
Cluster 2
P value | 0.0040
Immune system & inflammatory response
Activity (au)
Cluster 1
Cluster 2
P value | 0.0541
Keratinocytes & Proteolysis
Activity (au)
Cluster 1
Cluster 2
P value | 0.2631
Immune system & inflammatory response
Activity (au)
Cluster 1
Cluster 2
P value | 0.0441
Cellular adhesion & differentiation
Activity (au)
Cluster 1
Cluster 2
P value | 0.2845
Wnt pathway
Activity (au)
Cluster 1
Cluster 2
P value | 0.7453
Membrane transport
Activity (au)
Cluster 1
Cluster 2
P value | 0.7577
Transcription
Activity (au)
Cluster 1
Cluster 2
P value | 0.4819

C)

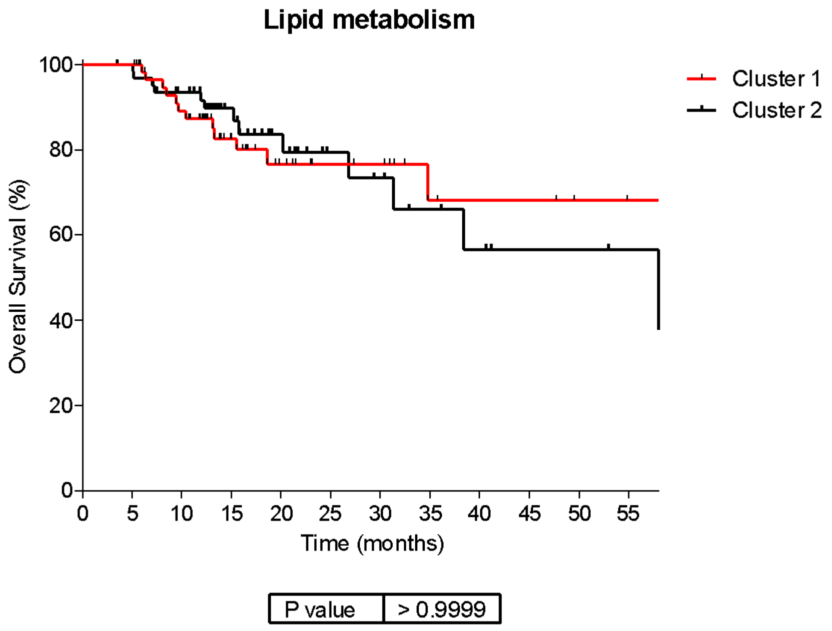
Lipid metabolism
Overall Survival (%)
100
80
60
40
20
0
0
5
10
15
20
25
30
35
40
45
50
55
Time (months)
Cluster 1
Cluster 2
P value
> 0.9999

Supplementary Table S1. Clinical characteristics of TCGA's selected gastric adenocarcinoma patients.

Clinical characteristics	Group	N (%)
**Age; median (range)**	63 (30-90)	142 (100)
**Gender**	Male	95 (66.9)
	Female	47 (33.1)
**Chemotherapy schedule**	5-Fluorouracil	73 (51.4)
	Platinum	23 (16.2)
	5-Fluorouracil+Platinum	46 (32.4)
**Stage**	IA	1 (0.7)
	IB	10 (7.0)
	II	12 (8.5)
	IIA	13 (9.2)
	IIB	23 (16.2)
	IIIA	33 (23.2)
	IIIB	25 (17.6)
	IIIC	19 (13.4)
	NA	6 (4.2)
**TCGA molecular subtype**	CIN	44 (31.0)
	MSI	16 (11.3)
	GS	20 (14.1)
	EBV	8 (5.6)
	NA	54 (38.0)
**Molecular subtype after non-assigned tumors***	CIN	77 (54.2)
	MSI	27 (19.0)
	GS	29 (20.5)
	EBV	9 (6.3)

* Non-assigned tumors were classified into TCGA's molecular subtypes using centroids. Abbreviations: CIN: Chromosome instability; EBV: Epstein-Barr virus; GS: Genomically stable; MSI: Microsatellite-instability. NA: Non-assigned.

Supplementary Table S2. Genes comprising each of the functional nodes

	NODE 1	NODE 2	NODE 3	NODE 4	NODE 5	NODE 6	NODE 7	NODE 8	NODE 9	NODE 10	NODE 11	NODE 12	NODE 13
**Function**	**LIpid metabolism**	**Transcriptio-n**	**Membrane**	**Cellular adhesion and proteolysis**	**Keratinocy-es**	**Immune system (Inflammatory response)**	**Keratinocyt-e and proteolysis**	**No function**	**Immune system (Inflamma-tory response)**	**Cellular adhesion and differentia-tion**	**Wnt pathway**	**Transmem-brane transport**	**Transcriptio-n**
**ID GENES**	LHFPL3	HOXA11	AADAC	A4GNT	ADD2	C19orf59	A2ML1	ABCB5	ADAM6	ABCA6	AGXT	ADRA2C	ACTL8
	TM6SF2	HOXA11AS	ABCA12	ABCA8	ANKS1B	CCL20	ALDH3B2	ACSL6	AICDA	ABCA9	APLP1	AGXT	ALOX12B
	HAPLN4	HOXA10	ABCG5	ABI3BP	ANO5	CSF2	ANXA8	ACTA1	AIM2	ADAM22	ASCL1	ANO3	AMDHD1
	SI	CDH17	ABCG8	ACTC1	AP3B2	CSF3	ANXA8L2	ACTG2	ALOX15B	ADAMTS15	ASGR2	BIRC7	ANKRD1
	FABP1	GUCY2C	ABHD12B	ADAMTS8	ARC	CXCL1	AREG	ACTN2	AMPD1	ADAMTS16	BAMBI	BRSK2	ATP6V1B1
	FABP2	PHGR1	ABO	ADAMTSL3	ARHGEF4	CXCL3	BTBD16	ADAM33	ART3	ADRA1D	BHMT	C17orf55	B4GALNT4
	BTNL3	GPA33	ACE2	ADCY5	ARMC3	CXCL6	C19orf33	ADAMTS18	ASPA	AKR1E2	BRSK2	C1QL4	BEX5
	SLC7A9	HOXA13	ACSM3	ADCYAP1	ASPG	CXCR1	C1orf161	ADCY2	BEND4	ALOX15	C17orf55	CA4	CASKIN1
	SLC5A9	CDX1	ADH6	ADH1B	ASTN1	CXCR2	C2orf54	AGTR1	BLK	ALPI	C1orf61	CACNA1E	CDKN2A
	NAT2	MEP1A	ADRA2A	ADH1C	BCL11A	CYP4X1	C5orf38	AHNAK2	BMX	AMH	C21orf29	CACNA1I	CELF5
	SLC17A4	ZIC2	AGR2	ADRB3	BEX2	DAPL1	CAPN14	AKAP6	C13orf30	AOX1	CA4	CHST13	CYorf15A
	LRRC19	ZIC5	AGR3	AFF3	BMP3	EMR3	CDH16	AR	C14orf64	APOA1	CAMK2B	CKM	DDN
	CHP2	CDX2	AKR1B10	AKR1C1	BNC1	FCGR3B	CLCA2	ARMC4	C21orf62	APOB	CDH22	CLDN6	DDX3Y
	ABCC6P1	MYO7B	AKR7A3	AKR1C2	BNIPL	GPR109A	CLCA4	ATP1A2	C4orf7	APOD	CDK5R2	CLDN9	DLL3
	CYP3A4	NR1I2	ALDH3A1	ALDH1A1	C17orf93	GPR109B	CLCN1	ATP2B2	C8orf80	AS3MT	CELF3	DKK1	DLX3
	ALDOB	IYD	ALG1L	ALDH1A2	C1orf173	GSDMC	COL17A1	BARX1	CCL13	ASCL2	CHGA	DOC2A	DLX4
	RBP2	CYP4F12	ALOX12P2	ALDH1L1	C2orf65	HAPLN1	COL28A1	BMPR1B	CCL14	ATCAY	CHGB	EMID2	DLX5
	RNF186	CYP4F3	ALPP	ANGPTL1	C8G	HBA1	COX6B2	C10orf93	CCL18	BCHE	CNTFR	F10	DLX6
	PDZK1	SLC6A7	ALPPL2	ANGPTL7	C9orf169	HBA2	CPA4	C21orf34	CCL19	BEX1	COL11A2	F7	DLX6AS
	KRT20	MOGAT3	AMBP	ANK2	CADM2	HBB	CST6	C2orf40	CCL23	BMP7	CPLX2	FAM90A1	DMRT3
	MOGAT2	LRRC66	AMN	AQP2	CDH12	IL11	CYP24A1	C4A	CCL26	BMPER	DMKN	FLJ16779	DPF1

	ABCC2	HOXA9	ANKFN1	AQP5	CECR2	IL13RA2	DSG3	C6orf176	CCL7	C13orf38	DNAH2	FXYD2	DUSP15
	SLC3A1	TM4SF20	ANKRD20A3	ARHGDIG	CHRM1	IL17A	DUSP27	C8orf85	CCR7	C1orf88	EEF1A2	GABRR1	DUSP9
	C19orf69	A1CF	ANKS4B	ASB2	CHST9	IL1A	EDAR	CACNA2D3	CD19	C1orf95	F10	GJA3	EIF1AY
	FAM23A	PDZD3	ANXA10	ATP13A4	CLDN19	IL1RL1	EDN2	CASQ1	CD1A	C20orf103	F7	GJB7	EN2
	PKLR	FAM84A	ARL14	ATRNL1	CNFN	IL24	EGR4	CCDC136	CD1C	C5orf23	FEV	GPC5	FAM131C
	MEP1B	CLDN3	ARSE	BAI3	CNGB1	IL6	EREG	CCL11	CD1E	C5orf49	FGA	GPR143	FOXL2
	MTTP	YBX2	ATOH1	BHMT2	CNR1	IL8	FAM169B	CDHR1	CD207	C6orf168	FGF19	GPR98	GPR158
	C17orf78	CHST5	ATP10B	BMP5	CNTN3	INSC	FAM83A	CHRDL2	CD22	CACNA1B	FSTL4	GRB14	GSC
	GBA3	C17orf73	ATP2C2	C16orf89	CPA6	KIAA1199	FGFBP1	CHRM2	CD79A	CACNG4	FXYD2	H19	HOTAIR
	ANPEP	MUC2	AZGP1	C1QTNF7	CRABP2	LAMC3	FIBCD1	CHRNA3	CD79B	CALY	GABRA4	HCG4	HOXC10
	OSTBETA	ABP1	B3GALT5	C1orf186	CSMD1	MME	GBP6	CHRNB4	CHI3L1	CAMKV	GALNT14	HMGA2	HOXC11
	CYP4F2	TMEM82	B3GNT6	C20orf114	CSTA	MMP1	GJB3	CNGA3	CHI3L2	CASC1	GLDC	HORMAD1	HOXC13
	XPNPEP2	SHD	BAAT	C20orf200	CTCFL	MMP10	GJB4	CNN1	CHIT1	CCDC80	GNG4	IGDCC3	HOXC8
	LCT	SLC26A3	BARX2	C21orf88	CUX2	MMP12	GJB5	CNTN1	CHST8	CDH2	GRIN1	IGF2BP1	HOXC9
	ADH4	PRLR	BCAS1	C3orf15	CXorf48	MMP3	GPR115	CNTN2	CKMT2	CDKL2	HAP1	ISM2	HOXD10
	APOBEC1	KCNJ3	BCL2L15	C3orf57	CYP4B1	MT1A	HES2	COL21A1	CLEC10A	CDO1	HPCAL4	LOC100133545	IGF2BP3
	TTR	NUP62CL	BTNL8	C4orf31	DCDC2	MT1G	IRX2	COL29A1	CLEC17A	CECR7	IGFALS	LOC284551	IGFBP1
	MYOM3	SPINK4	C10orf81	C6	DGCR5	MT1H	ITGB6	CPEB1	CNR2	CELF4	INSM1	LOC401387	INHA
	C3orf32	NAT8B	C11orf41	C6orf105	DLK2	MT1M	KLK10	CPNE6	COL4A3	CES1	IRX3	LOC440173	KCNT1
	NR1H4	NAT8	C11orf86	C6orf155	DLX2	NRG1	KLK11	CPXM2	COL4A4	CHRDL1	IRX5	LRP2	KDM5D
	CEL	VNN1	C12orf27	C6orf186	DMRTA1	PI15	KLK12	CTNNA3	CR1	CHRM3	ITIH2	LY6K	KIAA0319
	NLRP6	TUBAL3	C12orf36	C7	DNAH14	PPBP	KLK13	CYP21A2	CR2	CILP	KCNG1	MED12L	KIAA1751
	SLC19A3	ANXA13	C14orf105	C8orf46	DNER	PROK2	KLK6	DAB1	CRIP3	CILP2	KCNK15	MKRN3	KISS1R
	SLC14A2	KLK1	C14orf115	CABP4	DSC3	S100A12	KLK7	DCX	CXCL10	CLDN11	KIAA1045	NKAIN4	KLC3
	TINAG	PODXL2	C14orf34	CACNA2D1	DSG1	S100A8	KLK8	DES	CXCL11	CLEC2L	KIF1A	NPFFR2	KLRG2
	SLC15A1	KLHL35	C19orf46	CADM3	DTNA	S100A9	KRT13	DIRAS1	CXCL13	COCH	KIF5A	NPW	LGSN
	FRMD1	ENTPD8	C19orf77	CAPN6	EGF	SALL1	KRT14	DLG2	CXCL9	COL10A1	KIRREL2	NRCAM	LMX1B
	SLC30A10	MAPK15	C1orf110	CASQ2	ELF5	SLC38A5	KRT15	DPEP3	CXCR2P1	COL11A1	LASS1	OTOF	LOC440905
	CCL25	GALNT8	C1orf125	CASR	ENDOU	SLC6A14	KRT16	DPP6	CXCR5	COL9A3	LOC284551	PIPOX	LOC647946
	OTC	HOXB8	C20orf56	CBLN1	ENPP5	TEX11	KRT17	DPT	DARC	COMP	LRRC26	PITX2	MAT1A

	SLC13A2	HOXB9	C2CD4A	CCDC129	EVPLL	TRPA1	KRT23	DUSP26	DHRS2	CRABP1	MAP7D2	PLAC1	MESP1
	SLC34A2	NOS2	C3orf55	CCKBR	EVX1		KRT4	ECEL1	DNAH8	CREB3L3	MYT1	PNMA3	PANX2
	CYP2W1	CASP5	C3orf67	CCL21	FAM133A		KRT5	FAT3	DNASE1L3	CRLF1	NAT8L	PNMA6A	PNMA5
	CPS1	TDRD5	C4BPA	CD177	FAM153A		KRT6A	FGF5	FAM129C	CST1	NEUROD2	PNMT	PPP4R4
	C11orf53	BCL2L10	C4BPB	CDH19	FAM155B		KRT6B	FLNC	FAM135B	CST2	NEUROG3	PPP1R14C	PRAME
	SLC38A11	MUC12	C6orf222	CHD5	FAM181B		KRT80	FOXP2	FCAMR	CST4	NFE2	PPP1R1C	PRKY
	UNC93A	WNK4	C6orf58	CHL1	FAM189A1		LEMD1	GAP43	FCER1A	CYP11A1	NKAIN1	PRSS21	PRODH
	CREG2	EDN3	C8orf47	CHODL	FAM46B		LGR6	GPM6A	FCER2	CYP1B1	NKD1	PRSS30P	RPS4Y1
	F5	ESYT3	C9orf122	CLDN2	FAT2		LOC100131726	GRIA1	FCRL1	CYS1	NKD2	QPRT	RSPO4
	NPY6R	CADPS	C9orf152	CLGN	FIGN		LOC100216001	GRIK3	FCRL2	DACT2	NOL4	RHBG	SCAND3
	CNDP1	JPH1	CA12	CNKSR2	FLG		LOC554202	GRIK5	FCRL3	DCLK1	NOTUM	SLC29A4	SCT
	CHRNA7	DQX1	CA2	CNNM1	FMN2		LOC728643	GRPR	FCRL4	DDX43	NPHS1	SLC30A2	SH2D5
	BTN1A1	ABCA13	CA8	CNTNAP3	FOXN1		LY6D	HAND2	FCRL5	DEPDC7	NRCAM	SLC44A5	SLC13A5
	GLYATL1	DNAH3	CA9	COL19A1	GABRB2		LYPD3	HIF3A	FCRL6	DIO3	ORM1	SLC5A12	SYCP2L
		CNTD2	CALB1	COL4A5	GALNT13		MSLN	HLF	FCRLA	DLGAP1	PAPPA2	SLCO1A2	TFAP2C
		ISX	CALB2	COL4A6	GJB6		MUC16	HPD	FLJ40330	DPEP1	PCOLCE2	SLIT1	TKTL1
		XDH	CALHM3	COL9A1	GLP1R		NOXO1	HRNBP3	FLT3	DRD1	PIPOX	SYT7	TMPRSS13
		UBXN10	CAPN13	CPA3	GPR27		NXF3	HSPB7	FXYD1	DRD2	PLCH2	TAF7L	TNNI3
		TRPM6	CAPN8	CRTAC1	GPR37		PADI1	IGFN1	GAD1	DSCR6	PLEKHG4B	TDRD9	TNNT1
		NPM2	CAPN9	CTNND2	GPR87		PAX9	IGSF9B	GAPT	DYNC1I1	PNMT	TEX15	UPK2
		AKR1C4	CATSPERB	CTSG	GRHL3		PLA2G2F	IL31RA	GFI1B	ECHDC3	PPP1R14C	TG	USP9Y
		HTR1D	CCK	CXCL14	HCG22		PLA2G4E	ISL1	GZMK	ELAVL3	PROX1	TNNC2	UTY
		MFSD6L	CCL14-CCL15	CXCL17	HEPHL1		PSORS1C1	JPH2	HAMP	ELAVL4	PRSS30P	TNS4	VAX2
		STK31	CCL15	CYP2B7P1	HS6ST2		RAET1L	KCNA1	HLA-DOB	EPHX4	QPRT	UPK3A	WDR66
		IL1RL2	CCL24	CYP2E1	HS6ST3		RHCG	KCNA6	HLA-DQA2	ERBB4	RIMBP2	ZNF114	XIST
		C2orf70	CCL28	DBC1	HSPB8		S100A2	KCNB1	HLA-DQB2	ESPNL	RIMS4		ZDHHC8P1
		TCAM1P	CDHR2	DCHS2	IGSF1		SCEL	KCNH2	HLA-DRB5	ETNK2	RUNDC3A		ZFR2
		NOX1	CDHR5	DGKB	IGSF11		SERPINB2	KCNJ12	HLA-DRB6	EYA4	SCG3		ZFY
		PCK1	CEACAM5	DMBT1	KIF6		SERPINB3	KCNMA1	HPSE2	F13A1	SEC14L5		ZNF556

		GUCY1B2	CEACAM6	DSCAML1	KNDC1		SERPINB5	KCNMB1	HS3ST2	FAM178B	SEZ6		
		GAL	CEACAM7	EFHC2	KRT1		SLCO1B3	KCNQ4	IDO1	FAM180A	SLC10A4		
		TRIM54	CES3	ENAM	LASS3		SPRR1B	KIAA1644	IFNG	FAM184A	SLC29A4		
		NANOS3	CFTR	ENPP3	LGALS7		SPRR3	KLF17	IGJ	FAM3B	SLC30A2		
		ACHE	CHST4	ENTPD3	LGALS7B		SULT2B1	KLHL13	IKZF3	FAM43B	SLC35F3		
		C6orf223	CIDEC	EPHA7	LGI3		SYT8	L1TD1	IL17REL	FAM5C	SLC38A3		
		TBX10	CLDN10	ESRRG	LOC154822		TGM3	LDB3	JSRP1	FGF14	SMOC1		
		DACH1	CLDN18	EYA2	LPAR3		TNFRSF6B	LGALS12	KCNJ5	FGF9	SST		
		CNTNAP2	CLIC6	FABP4	LRAT		TPRXL	LMO3	KCNQ5	FGL1	SYT5		
		PIWIL1	CLRN3	FAM150A	LRRTM1		TRIM29	LMOD1	KEL	FMO1	SYT7		
		NMU	CMBL	FAM163A	LY6G6C		UCA1	LOC146336	KIAA0125	FMO2	TCL6		
		EMX1	COL22A1	FAM198A	LYNX1		UNC5A	LOC283174	KLHL14	FNDC1	TH		
		DMBX1	CP	FBLN1	MAL		UPK3B	LOC728264	LILRA4	FNDC5	TMEM151A		
		AQP12B	CRYM	FGF10	MGAT5B		WNT10A	LRRC10B	LOC286002	FOSB	TMEM63C		
		FOXJ1	CTSE	FHL1	MLF1			MAB21L2	LOC400696	FOXD3	TNFRSF19		
		RANBP17	CYP2B6	FIGF	MPV17L			MAPK4	LOC96610	FREM1	TNNC2		
		TMEM229A	CYP2C18	FLJ42875	MYEF2			MAPT	LPPR1	FZD10	TPH1		
		FAM55B	CYP2C19	FREM2	MYO3A			MKX	LTF	GABBR2	TREML2		
		MYADML2	CYP2C9	FRMPD1	NAP1L2			MYH11	LY9	GABRE	TRIM72		
		PRSS33	CYP2D6	FST	NCCRP1			MYLK	MARCO	GAL3ST2	TRPV3		
		GABRA2	CYP3A5	FUT9	NIPAL4			NACA2	MGC29506	GALNTL1	TUBB2B		
		HOXB13	DDC	GABRB3	NOX5			NBLA00301	MMRN1	GAS1	TUBB4		
		C11orf20	DEFB1	GALNT9	NPSR1			NEFM	MS4A1	GDF6	UCHL1		
		FEZF1	DHRS9	GATA5	NPY1R			NELL1	MYBPC2	GFRA3	UPB1		
		CYP4F11	DMRTA2	GDF10	NTS			NGFR	NCF1B	GLP2R	UPK3A		
		PLA2G4D	DNAH5	GFRA1	OCA2			NKAIN2	NEURL3	GLT25D2	VGF		
		ARX	DPCR1	GHRL	ODZ1			NKX3-2	NLRP2	GLTPD2	VWA5B2		
		CXCL5	DUOX1	GIF	PADI3			NPAS4	NLRP7	GNAO1	WNT11		
		LOC286467	DUOX2	GPR133	PAK7			NRG3	NXPH4	GPC3			
		PLA2G2A	DUOXA1	GPR15	PCDH20			NRXN1	P2RY12	GPR1			

		TREH	DUOXA2	GREM2	PCLO			NRXN3	PAX5	GPR120			
		LOC100130238	EFNA2	GRIA4	PCP4L1			NUDT10	PCDH11X	GPR81			
		FAM66D	ELFN2	GRIN2A	PKD1L2			NXPH3	PCP4	GREB1L			
		FABP6	ENTPD2	GSTA1	PKP1			ODZ2	PKHD1L1	GREM1			
			EPHX3	GSTA2	PON3			PART1	PLA2G2D	GRM4			
			EPN3	HABP2	PP14571			PCA3	PLD4	GRP			
			EPS8L3	HHIP	PPP1R3C			PCDH10	PNOC	GTSF1			
			ERN2	HLA-G	PPP1R9A			PCDH9	POU2AF1	GUCA1A			
			ESPN	HOMER2	PPP2R2C			PCYT1B	RGS13	GYLTL1B			
			FAM101A	HTR3A	PRKAA2			PDZRN4	RPPH1	HAS1			
			FAM132A	IGSF10	PRSS27			PEG3	SELP	HCN4			
			FAM177B	IL33	RAET1G			PENK	SIGLEC14	HMP19			
			FAM189A2	IP6K3	RBM11			PHYHIPL	SLC1A2	HOXA7			
			FAM3D	ITGA8	RGS6			PLIN4	SPIB	HP			
			FAM95B1	ITIH4	RIBC2			PLN	SPP1	HRASLS			
			FBP2	KBTBD12	RIC3			PNMAL1	STAP1	HRASLS5			
			FBXO2	KCNA2	RPRM			PRPH	SUCNR1	HS3ST5			
			FCGBP	KCNA5	RPS28			PRRT4	TACR1	HSD3B2			
			FER1L4	KCNE2	SCML2			PSD	TCL1A	HSPB6			
			FER1L6	KCNH8	SCNN1B			PTCHD1	TLR10	HTR2A			
			FLRT3	KCNIP1	SFTPB			PYGO1	TNFRSF13B	HYDIN			
			FOLR1	KCNJ15	SH3GL2			RBM24	TNFRSF13C	IBSP			
			FOXA1	KCNJ16	SIX3			RET	TNFRSF17	IGF1			
			FOXA2	KCNK2	SLC13A3			RGMA	UBD	IGF2			
			FOXD1	KIAA2022	SLC24A5			RNF150	VPREB3	IGFBPL1			
			FRAS1	KIRREL3	SLC26A7			RTN4RL1	ZNF385D	IGFL2			
			FUT3	KY	SLC27A6			RYR2	ZNF683	INA			
			FUT6	LCN10	SLC30A3			RYR3		ISM1			
			FXYD4	LEFTY2	SMC1B			SCN2B		ITGBL1			
			GABRP	LIPF	SNAP25			SCUBE2		ITIH3			
			GALNT5	LIX1	SYT9			SHISA9		JPH3			

			GATA4	LOC100128164	TAC3			SIX2		KANK4			
			GC	LOC220594	TBX4			SLC8A2		KCNG3			
			GCKR	LOC284233	TDRD1			SLITRK2		KCNH3			
			GCNT3	LOC643763	TGM1			SLITRK4		KCNK3			
			GDA	LONRF2	THNSL2			SLITRK5		KERA			
			GDPD2	LPHN3	TMEM213			SMOC2		KIAA1324L			
			GGT6	LUZP2	TP63			SNORD15B		KLF15			
			GJB1	MADCAM1	TRPV6			SORBS1		L1CAM			
			GKN1	MAMDC2	TTLL7			SOX8		LAMA1			
			GKN2	MAOB	ULBP1			SPEG		LBP			
			GOLT1A	MASP1	ULBP2			SPON1		LGR5			
			GP2	MS4A2	UPK1A			SPTBN4		LHFPL4			
			GPD1	MSMB	VWC2			SSTR5		LIPC			
			GPR110	MTUS2	WDR17			STAC		LMOD1			
			GPR128	MUC6	WDR87			SYNC		LOC145837			
			GPT	MUSK	ZNF492			TACR2		LOC162632			
			GPX2	MYO3B	ZNF676			TCEAL2		LOC255167			
			GSTT1	MYOC	ZNF750			TFPI2		LOC441666			
			HAR1B	MYOCD	ZNF98			TPSG1		LOC644172			
			HAS3	MYOM1				UNC45B		LOC728819			
			HAVCR1	MYRIP				VIP		LOC731789			
			HEPACAM2	NBEA				WNT9A		LPPR3			
			HGD	NCAM1				ZFP57		LRP4			
			HHLA2	NDP				ZNF257		LRRC15			
			HKDC1	NFASC				ZNF334		LRRC43			
			HMGCS2	NLGN1				ZNF385B		LRRC4C			
			HNF1B	NOVA1				ZNF727		LRRIQ1			
			HNF4A	NRG2				ZNF835		LRRN1			
			HPGD	NTNG1						LRRN4			
			HPN	OLFM4						MAEL			
			HRASLS2	P2RX2						MCOLN3			
			HSD17B2	PAR5						MEOX2			
			IHH	PCDH19						MFAP5			
			IL17C	PCDHA4						MGP			

			IL1R2	PCDHAC1						MMP11			
			IL1RN	PCDHAC2						MMP13			
			ITLN1	PCSK1						MS4A15			
			KCNK10	PCSK2						MSI1			
			KCNK9	PDE1C						MST1P9			
			KCNS1	PDIA2						MYO18B			
			KIAA1239	PF4						MYOZ3			
			KIAA1324	PGA3						NALCN			
			KISS1	PGC						NAV3			
			KLHDC7A	PGM5						NCAM2			
			KPNA7	PLCXD3						NEBL			
			KRT7	PLIN1						NEFL			
			LCN2	PLP1						NEK5			
			LEFTY1	PLXNB3						NEU4			
			LGALS2	PMP2						NOG			
			LGALS4	PPARGC1A						NOS1			
			LGALS9B	PPP1R1A						NPR3			
			LGALS9C	PPYR1						NPTX1			
			LIPH	PRIMA1						NPTXR			
			LOC100124692	PRSS1						NRK			
			LOC100127888	PRUNE2						NRXN2			
			LOC201651	PTCH2						NSUN7			
			LOC284578	PTPLA						NTRK2			
			LOC284749	PTPN5						NTRK3			
			LOC389332	PTPRT						NTSR1			
			LOC553137	PTPRZ1						ODZ3			
			LOC84740	PYGM						OGDHL			
			LOC93432	REEP1						OGN			
			LRRC31	RELN						OMD			
			LTK	RGPD7						OR7D2			
			LYPD2	RHOV						PAK3			
			LYPD6	RIMS1						PCDH8			
			LYPD6B	RNF165						PCDHA11			
			LYZ	ROBO2						PCDHA12			

			MAPK8IP2	RPS6KA6						PCDHB5			
			MB	RSPO2						PCDHB6			
			MFSD4	SCARA5						PCDHB8			
			MGAM	SCGB3A1						PCSK1N			
			MIA	SCN7A						PDGFRL			
			MIA2	SCUBE1						PDZD4			
			MIOX	SDR42E1						PEG10			
			MLPH	SEMA3E						PHACTR3			
			MLXIPL	SFRP1						PI16			
			MMEL1	SFTPA2						PIRT			
			MMP7	SGCA						PLXNA4			
			MRAP2	SHISA3						PNCK			
			MS4A8B	SIGLEC6						POPDC3			
			MTNR1A	SLC16A9						PPAPDC1A			
			MUC1	SLC26A9						PPFIA2			
			MUC13	SLC2A4						PRELP			
			MUC17	SLC38A4						PRG4			
			MUC20	SLC9A2						PRND			
			MUC4	SLC9A3						PROC			
			MUC5B	SLC9A4						PRR18			
			MYBPC1	SLCO4C1						PTGIS			
			MYCN	SLITRK6						PTH2R			
			MYO1A	SNAP91						PTPN20B			
			MYPN	SNORD116-4						PTPRN			
			NEB	SORCS1						RAB3C			
			NMUR2	SOSTDC1						RASSF10			
			NPC1L1	SOX10						RBP4			
			NR0B2	SOX2						RBPMS2			
			NRG4	SOX21						RERGL			
			NWD1	STK33						RGN			
			ODAM	SUSD4						RGS7			
			ONECUT2	SYCP2						RGS9			
			ONECUT3	SYNM						RIMS2			
			OVOL1	SYNPO2						RND2			
			PALM3	SYT13						RNF126P1			
			PCDP1	TCN1						RNF182			
			PCSK9	TESC						RNF212			

			PDE11A	TFCP2L1						RSPO1			
			PDE4C	TMEM100						RSPO3			
			PDZK1IP1	TMEM132C						SALL4			
			PI3	TMEM61						SCN2A			
			PIGR	TMPRSS3						SCN5A			
			PIK3C2G	TPO						SCRG1			
			PKHD1	TPSB2						SDK2			
			PLA2G10	TPSD1						SEMA3D			
			PLA2G3	TRHDE						SEZ6L			
			PLA2G4F	TRIM50						SFRP2			
			PLAC8	TYRP1						SFRP4			
			PLEKHB1	VIPR2						SFRP5			
			PNPLA3	VIT						SFTPD			
			POU2F3	WSCD2						SHISA6			
			PPP1R14D	WT1						SIX1			
			PPP1R1B	XKR4						SLC35D3			
			PRAP1	ZNF536						SLC4A3			
			PRKCG	ZNHIT2						SLC6A4			
			PRKG2	ZPLD1						SLC7A14			
			PROM1	ZSCAN23						SLC7A2			
			PROM2							SLC7A4			
			PRSS22							SLIT2			
			PRSS3							SLITRK3			
			PSAPL1							SLN			
			PSCA							SMTNL2			
			PTPRR							SNCAIP			
			RAB3B							SOHLH2			
			RAP1GAP							SP5			
			RASAL1							SPESP1			
			RDH12							SPOCK1			
			REG1A							SRPK3			
			REG3A							ST6GAL2			
			REG4							STMN2			
			RGL3							STRA6			
			RHBDL2							SULT4A1			
			RNF183							SVOP			
			RORC							SYN2			

			ROS1							SYN3			
			RTBDN							SYNPR			
			S100A14							SYT1			
			S100P							SYT3			
			SAA1							SYT4			
			SAA2							TBX18			
			SAA4							TDGF1			
			SCIN							TDGF3			
			SCNN1A							TERT			
			SCTR							TF			
			SDR16C5							THBS4			
			SEC14L4							THPO			
			SEMG1							THSD7B			
			SERPINA1							TMEFF2			
			SERPINA3							TMEM130			
			SERPINA4							TMEM35			
			SERPINA5							TMEM59L			
			SERPINB7							TMPRSS5			
			SFTA2							TNFRSF11B			
			SGK2							TNN			
			SHH							TPD52L1			
			SLC22A3							TRPM5			
			SLC28A2							TTPA			
			SLC28A3							TTYH1			
			SLC39A5							UGT3A2			
			SLC44A4							UNC80			
			SLC4A4							UTS2			
			SLC5A1							UTS2R			
			SLC5A5							VSTM2L			
			SLC6A19							VTN			
			SLC6A20							VWA2			
			SLPI							WIPF3			
			SPAG17							WISP2			
			SPDEF							WISP3			
			SPDYC							WNT2			

			SPINK1							XG			
			SPINK5							XIRP1			
			SRMS							ZBTB16			
			SSTR1							ZNF541			
			ST6GALNAC1							ZNF883			
			STX19							ZYG11A			
			SULT1A2										
			SULT1B1										
			SULT1C2										
			SULT1E1										
			SYT12										
			SYTL5										
			TACSTD2										
			TEX19										
			TFF1										
			TFF2										
			TFF3										
			TFR2										
			TLX1										
			TM4SF4										
			TM4SF5										
			TMEM189-UBE2V1										
			TMEM195										
			TNNC1										
			TNNT2										
			TRIM31										
			TRIM40										
			TRIM7										
			TSPAN1										
			TSPAN8										
			TTLL6										
			TUBBP5										
			UGT1A1										
			UGT1A10										
			UGT1A6										
			UGT1A9										
			UGT2A3										
			UGT2B15										
			UGT2B7										

			UNC5CL										
			UPK1B										
			USH1C										
			VEPH1										
			VGLL1										
			VIL1										
			VNN3										
			VSIG1										
			VSIG2										
			VSNL1										
			VTCN1										
			VWA3B										
			WDR72										
			WFDC2										
			WNT7B										
			ZBTB7C										
			ZG16B										

Supplementary Table S3. Lists of genes in which each layer classification is based on.

CML	Lipid metabolism	Proteolysis layer
XIST	OLFM4	LIPF
MUC2	RPS4Y1	PGA3
CHGA	FABP1	REG1A
IGF2BP1	SPRR3	GKN1
C4orf7	KRT13	C20orf114
APOB	SI	PGC
DKK1	GSTT1	REG3A
DES	KRT6A	MUC6
THBS4	DDX3Y	GKN2
CLDN6	KRT4	TFF2
SLC34A2	DUSP27	KRT20
PRAME	DMBT1	REG4
APOA1	KRT5	DPCR1
PHGR1	KDM5D	MSMB

C7	MUC17	CXCL17
CR2	CXCL5	ANXA10
PCSK1N	KRT6B	CLDN18
NPSR1	SPINK4	C6orf58
PON3	CDX1	MUC5B
KIF1A	DSG3	AQP5
WFDC2	MEP1A	AKR1B10
C2orf40	GP2	FER1L6
SCRG1	LY6D	PSCA
ADH1B	SPRR1B	VSIG1
MS4A1	CCL25	DUOX2
PKP1	SLC26A3	LTF
COMP	ALPPL2	DUOXA2
EEF1A2	TM4SF20	ITLN1
HAND2	CPS1	TFF1
HPN	GABRP	PIGR
SST	LYPD2	CTSE
DACT2	SLC6A19	FUT9
BMP3	CHP2	TCN1
SOX2	TM4SF4	UPK1B
NBLA00301	HOXC10	B3GNT6
TF	IRX2	SLC9A4
BEX1	KLK6	A4GNT
OGDHL	PIK3C2G	SOX21
CACNG4	SCEL	CA9
TCL1A	NOTUM	ADH1C
VIP	KRT17	SLC5A5
CAPN6	RHCG	VSIG2

ATP1A2	SLC9A3	CAPN9
CRABP1	HMGCS2	FAM177B
PNCK	UGT2A3	LGALS9C
SMOC1	SLC28A2	CA2
HOXC13	MUC16	
CHRM2	SERPINB2	
CHRDL1	PRSS1	
B4GALNT4	KLK11	
LGSN	GIF	
CCL19	PI3	
NPTX1	LOC84740	
PPP1R1A	KLK7	
OGN	KLK10	
CNTN1	GATA5	
MAPK4	KRT16	
LOC440905	NLRP2	
FGF19	MMP3	
PENK	CYP2W1	
MYH11	PRSS21	
MYOC	MTTP	
PI16	FOXJ1	
SCN7A	PIWIL1	
PAX5	A2ML1	
CNTFR	KRT14	
PDZRN4	ZFY	
ABCA8	SAA1	
SFRP2	CDH17	
FCRL1	FABP2	

TCEAL2	SLC13A2	
GPA33	UTY	
PRKAA2	CEACAM7	
CHGB	NPC1L1	
CASQ2	KRT23	
PCLO	PLA2G2A	
XPNPEP2	MMP7	
TEX15	HABP2	
PPP2R2C	KLK13	
FREM1	GUCY2C	
GLDC	SLC15A1	
DLX3	USP9Y	
NCCRP1	PRKY	
TACR2	COL11A1	
MAL	GAL	
BLK	DPEP1	
PLP1	RBP2	
PCDH10	ALDOB	
FCER2	PCK1	
COL4A6	KLK8	
LRRN1	FGFBP1	
PCP4L1	GPR128	
ACTN2	FGA	
ACTG2	MSLN	
SFRP5	SERPINB3	
DPP6	ADH4	
GFRA3	MUC4	
FAM129C	ONECUT3	

PRIMA1	HEPACAM2	
FCRLA	EIF1AY	
L1CAM	PPP1R1B	
CNR1	FOLR1	
HSPB6	GPR110	
CHRDL2	GSTA1	
APOD	ANXA8	
NRK	ORM1	
CHST9	PRAP1	
BEX2	TKTL1	
VTN	MYBPC1	
CADM3	GC	
RSPO4	ANXA13	
WSCD2	SULT1E1	
SIX2	CLCA2	
NGFR	NMUR2	
CILP	CDHR2	
LRP2	VGLL1	
SLC8A2	NOX1	
C16orf89	CLCA4	
SEC14L4	UCA1	
SYNM	TRIM54	
CTNND2	ZIC2	
PLIN4	SAA2	
MAMDC2	CEACAM5	
AGTR1	ISX	
HSPB7	ACTL8	
FAM189A1	SERPINA4	

PEG10	UGT1A6	
UPK3A	TTR	
RIMS1	SERPINB7	
SLITRK5	ATP13A4	
SLC7A4	CLDN2	
ADCY5	SLCO1B3	
CES1	WDR72	
SFRP1	LGALS7B	
GREM2	CHST5	
SCARA5	MIA	
SYNPO2	VTCN1	
SHISA3	BTNL3	
CNN1	CYorf15A	
DNER	SLC26A9	
ODZ2	CDH16	
C1orf95	GGT6	
MSI1	LEFTY1	
ECEL1	MAP7D2	
NRXN1	RBP4	
SLC13A5	CREB3L3	
OMD	ALDH3B2	
KCNB1	SLC6A14	
EPHA7	UGT1A10	
FOXP2	CEACAM6	
CD19	ALPP	
CRTAC1	FAM83A	
MADCAM1	GABRB3	
GFRA1	FAM3D	

LDB3	PDIA2	
SOX10	ALPI	
IGFBPL1	OTC	
FMN2	GJB5	
VPREB3	CYP3A4	
PLCXD3	FAM3B	
PRUNE2	CYP2B6	
SLC38A3	TNNT1	
KIAA0125	NR1I2	
GNG4	CSF3	
PTCHD1	DCDC2	
FAM155B	ZIC5	
ANGPTL1	C4BPA	
CLEC17A	CST1	
FSTL4	HORMAD1	
BCHE	KRT7	
CDH19	CDX2	
GRIN2A	ABCA12	
SLIT2	LBP	
LONRF2	TGM3	
PGM5	TFF3	
COL4A3	ANPEP	
DARC	MOGAT2	
CNKSR2	CA4	
RIMBP2	FCGBP	
COL19A1	WNT11	
CACNA1E	C20orf56	
PCDH20	PCP4	

HRASLS5	SLC7A9	
FCRL2	MUC12	
CRLF1	SLC39A5	
GRIK3	CALB1	
C1orf88	NOS2	
ZBTB16	GBP6	
HS6ST3	CEL	
DSCR6	KCNJ3	
ADAMTS8	SLC17A4	
HS6ST2	UGT2B15	
VIPR2	AADAC	
HIF3A	CLDN10	
SLC30A2	LOC389332	
PART1	HOXB13	
STMN2	TMEM189-UBE2V1	
THSD7B	NXF3	
PNMA3	BAAT	
FABP4	FIBCD1	
CLEC2L	EMX1	
PCSK2	CYP4F2	
DCHS2	C19orf46	
GPR27	FREM2	
KCNK2	SFTA2	
KCNMA1	LOC100124692	
PTGIS	SLC44A5	
SNAP25	GJB6	
PCDH9	SOSTDC1	
ASTN1	LOC731789	

FLNC	PROM1	
CD22	ALDH1L1	
CHI3L2	HHLA2	
NEFL	PSAPL1	
AOX1	SYT8	
FLJ40330	LCN2	
RGN	CFTR	
DCX	TRIM29	
SGCA	KBTBD12	
NIPAL4	SCGB3A1	
CNR2	ZFP57	
DNASE1L3	TNS4	
SPEG	UGT1A1	
RIMS4	WISP3	
C6orf186	ERN2	
SHISA9	A1CF	
PCOLCE2	DSC3	
CD79A	UNC93A	
RELN	ACE2	
TMEM59L	SFRP4	
PHYHIPL	MYO1A	
C6orf168	C14orf105	
ADCYAP1	LGR5	
KCNK3	LY6K	
FMO2	SULT4A1	
KLHL14	HOXA11	
LEFTY2	GPR143	
TNFRSF13B	LYPD6B	

C20orf200	DAPL1	
TYRP1	PITX2	
AP3B2	MYO7B	
KIAA2022	SPINK1	
FCRL5	HLA-DRB6	
SMTNL2	FUT6	
PLN	LGALS9B	
NOVA1	CTCFL	
RGMA	ZNHIT2	
FCER1A	TRPV6	
NOS1	GTSF1	
CCL21	EN2	
LRRIQ1	MYO18B	
KIF5A	SLC3A1	
CXCR5	RPS28	
LRRTM1	PADI1	
ITGA8	EYA2	
DPT	ODAM	
WISP2	SDR16C5	
NLGN1	LGALS7	
ZYG11A	UPK3B	
TMEM35	SEMA3E	LIPF
FCRL3	AQP2	PGA3
MYOCD	CAPN13	REG1A
MEOX2	HAVCR1	GKN1
MED12L	NAT2	C20orf114
SORCS1	PTPRZ1	PGC
NRXN3	CYP24A1	REG3A

IGDCC3	EREG	MUC6
RYR2	B3GALT5	GKN2
RSPO2	SERPINB5	TFF2
CLGN	LOC553137	KRT20
PSD	CLDN3	REG4
POPDC3	ADH6	DPCR1
MYRIP	CADPS	MSMB
ISM1	DMKN	CXCL17
SLC4A3	SLC35D3	ANXA10
ATRNL1	COL10A1	CLDN18
SCG3	LMX1B	C6orf58
SNAP91	SLC6A20	MUC5B
TMEM100	DLX6	AQP5
GPM6A	APOBEC1	AKR1B10
ODZ1	DUSP9	FER1L6
LMOD1	ANXA8L2	PSCA
CADM2	FUT3	VSIG1
LRAT	SPINK5	DUOX2
ABCA9	CHIT1	LTF
TLR10	IHH	DUOXA2
MUSK	HOXA13	ITLN1
STAP1	INSM1	TFF1
CNTN2	DHRS2	PIGR
CHD5	CHST4	CTSE
FAT3	ALDH3A1	FUT9
ANGPTL7	TRPA1	TCN1
ASGR2	GSTA2	UPK1B
LOC643763	ITIH2	B3GNT6

CDO1	ENTPD8	SLC9A4
TUBB4	SCTR	A4GNT
RIC3	SULT1C2	SOX21
RPRM	MLXIPL	CA9
BMPR1B	MS4A8B	ADH1C
NPY1R	MMP12	SLC5A5
PRELP	SYT12	VSIG2
NXPH3	LOC723809	CAPN9
AFF3	PADI3	FAM177B
KCNA6	ROS1	LGALS9C
PTPRT	KCNJ16	CA2
NAT8L	C5orf38	
ADAM33	IGFBP1	
LMO3	TFAP2C	
LOC255167	F7	
NPR3	LOC93432	
GALNTL1	GATA4	
BAI3	ABCC2	
C21orf34	CP	
GRIK5	SPDEF	
WDR17	VWA2	
NTRK3	LGR6	
IGSF11	PPP1R9A	
ADCY2	PLA2G2F	
GALNT13	DEFB1	
C8orf85	MEP1B	
TMEM63C	HP	
TACR1	GREB1L	

DLG2	RNF186	
ADD2	MMP10	
HPSE2	KCNA1	
SELP	LOC146336	
JPH3	WNT7B	
CPEB1	EDN3	
KIAA1324L	GALNT14	
ABI3BP	ATP10B	
RSPO3	TINAG	
CYP1B1	SPIB	
TUBB2B	S100A8	
NBEA	PTPRN	
KIF6	HRASLS2	
ITIH3	SLC19A3	
CR1	C8G	
SLC2A4	TSPAN8	
PYGM	GBA3	
HYDIN	NR1H4	
PAK3	NTS	
CELF4	PCDP1	
JPH2	C11orf86	
BMPER	C6	
SEMA3D	AZGP1	
TMEM132C	SERPINA3	
PNOC	C6orf105	
TPO	GPC3	
SCN2B	TDRD9	
KCNH2	WT1	

GAP43	HLA-DQA2	
FOXD3	TPRXL	
PKHD1L1	SCNN1B	
RSPO1	GABRB2	
MAPT	PKHD1	
GNAO1	C1orf125	
REEP1	GPR87	
ZNF676	FGL1	
IGSF1	TDRD5	
RERGL	C4BPB	
KLF15	CCL20	
IGF1	SYNPR	
CD1C	SOHLH2	
KLF17	ESRRG	
SCN5A	KCNE2	
SRPK3	LCT	
LCN10	GABRR1	
RBM24	DUOXA1	
SNCAIP	STK31	
ADRA1D	CST6	
ZNF385D	HRNBP3	
LOC728264	SAA4	
RUNDC3A	ELF5	
CHRM3	GAD1	
ASB2	PRKCG	
SYN3	EDAR	
NRXN2	KIRREL2	
C8orf46	IGFALS	

DUSP26	MOGAT3	
DTNA	BTNL8	
VIT	ASPG	
BEND4	HOXD10	
CD79B	DSCAML1	
LASS1	CDHR1	
ZNF536	MMP13	
FCRL4	VSNL1	
C1QTNF7	HOXA11AS	
CCL14	PCDHA11	
MYEF2	SYTL5	
NKAIN1	MKRN3	
C13orf30	IL24	
COL4A4	FEZF1	
RND2	GALNT8	
UCHL1	ZNF750	
AR	SEMG1	
PTPN5	UGT1A9	
KIAA1045	TRHDE	
NRG2	TMPRSS13	
DYNC1I1	NEUROG3	
MLF1	SYN2	
NCAM1	GJB7	
ABCA6	KCNH8	
P2RY12	CHRNA3	
CHRNB4	NEB	
PNMAL1	PCDHA12	
MMRN1	NTSR1	

AS3MT	KLRG2	
FAM181B	AGR2	
FAM184A	CYP4F11	
RNF150	FCAMR	
KCNG1	TRPM6	
NAP1L2	LRRC19	
RBPMS2	MT1H	
PDZD4	GLTPD2	
FLT3	SCN2A	
BHMT2	PPAPDC1A	
LYNX1	SYT13	
PAR5	SYT1	
KCNIP1	CALB2	
PLXNA4	TMEM195	
RGS9	GPR98	
HSPB8	KCNK15	
CLEC10A	CTNNA3	
AKAP6	IYD	
CYP11A1	MAT1A	
PCDHB5	S100A2	
ANK2	CST4	
ANKS1B	IGF2BP3	
KCNQ5	C19orf77	
XKR4	CELF3	
FHL1	IGJ	
SIGLEC6	PPP1R14C	
DCLK1	SULT1B1	
LRRC10B	YBX2	

NPTXR	TNNI3	
ZNF727	ARHGDIG	
RNF165	NPW	
PCYT1B	NEU4	
NFASC	KLK1	
CD1E	COL17A1	
HPCAL4	OCA2	
C21orf62	PRPH	
LRRC43	C6orf223	
CCR7	TUBBP5	
FNDC5	GDPD2	
NUDT10	FBP2	
PDE1C	GDA	
FBLN1	PRODH	
ADAMTSL3	CLRN3	
MYOZ3	NKD1	
PYGO1	GAL3ST2	
ETNK2	HOXB8	
FXYD1	PHACTR3	
GAPT	CD177	
MS4A2	SIX3	
	RASSF10	
	C3orf57	
	COL9A3	
	PLA2G2D	
	GJB4	
	FABP6	
	FRMD1	

	CNTNAP2	
	DDC	
	VIL1	
	CYP2C18	
	PROM2	
	NRG1	
	TRIM31	
	MMP1	
	COL9A1	
	PLA2G3	
	GRP	
	PTPN20B	
	HOMER2	
	ACTC1	
	TUBAL3	
	LOC644172	
	CCKBR	
	PNMA5	
	LGALS4	
	CIDEC	
	SLC7A14	
	FAM189A2	
	RNF182	
	FOXD1	
	MS4A15	
	COL11A2	
	ABCG8	
	LHFPL3	

	SPAG17	
	HOXA10	
	HOXB9	
	FAM178B	
	FAM133A	
	CPLX2	
	HTR3A	
	UNC5A	
	GPR115	
	SFTPB	
	ST6GALNAC1	
	CPA4	
	GPR158	
	MARCO	
	H19	
	MT1M	
	LRRC31	
	CAPN14	
	S100A9	
	SERPINA5	
	UGT2B7	
	CNTD2	
	ANKRD1	
	CYP2B7P1	
	HTR1D	
	RGL3	
	AMBP	
	ITGBL1	

	C14orf34	
	PLXNB3	
	UPK1A	
	IL17REL	
	ZDHHC8P1	
	KIAA1324	
	CYP4B1	
	IGF2	
	CCK	
	KLK12	
	FXYD4	
	C1orf110	
	NR0B2	
	CXCL6	
	TFPI2	
	LIPC	
	AGR3	
	DMBX1	
	SLITRK6	
	SYT5	
	NMU	
	BRSK2	
	TCL6	
	IL11	
	KNDC1	
	ABHD12B	
	KRT15	
	S100P	

	GPR109A	
	ALDH1A2	
	XG	
	HHIP	
	EDN2	
	DDX43	
	IRX3	
	FBXO2	
	VNN1	
	ANO3	
	TMEM229A	
	CDKN2A	
	PPP1R14D	
	LASS3	
	CKMT2	
	INHA	
	NKAIN2	
	ATOH1	
	ARX	
	GRHL3	
	CSMD1	
	LGALS2	
	FAM131C	
	BARX1	
	DLL3	
	MAEL	
	BMP7	
	JSRP1	

	TNNC1	
	TBX10	
	MUC13	
	CATSPERB	
	CACNA1B	
	KCNS1	
	SSTR1	
	CCL26	
	NLRP7	
	TPSD1	
	CYP2C9	
	OSTBETA	
	CNDP1	
	BNC1	
	SCAND3	
	CNNM1	
	SSTR5	
	C17orf78	
	LEMD1	
	PCDHAC2	
	VNN3	
	PTH2R	
	TM6SF2	
	SLC6A4	
	VSTM2L	
	PROX1	
	CYP2C19	
	C8orf47	

	ADAM6	
	NELL1	
	ELFN2	
	SALL4	
	FCGR3B	
	UTS2	
	C12orf36	
	AKR7A3	
	SLC5A1	
	ANO5	
	CA8	
	NKX3-2	
	C3orf55	
	KANK4	
	HKDC1	
	MST1P9	
	GCKR	
	PLEKHG4B	
	RGS13	
	PKLR	
	LTK	
	ARSE	
	ABCA13	
	PAX9	
	ZNF114	
	ZG16B	
	MYT1	
	EPHX3	

	SLC9A2	
	CXCL13	
	SLC38A4	
	PNMA6A	
	CPNE6	
	MYOM3	
	MGAT5B	
	ADRB3	
	IL17C	
	PNPLA3	
	TMEM213	
	DMRTA2	
	HOTAIR	
	RASAL1	
	ADRA2A	
	ALOX12B	
	CLDN19	
	GJB1	
	CPA6	
	PPBP	
	TTYH1	
	VWA5B2	
	SFTPA2	
	LOC145837	
	SLC5A12	
	GRPR	
	SLC38A11	
	WNK4	

	TNFRSF17	
	CAMKV	
	BCAS1	
	IGFL2	
	PCSK9	
	PLA2G4F	
	SYT7	
	CREG2	
	RPS6KA6	
	CYP4F12	
	SALL1	
	SYT4	
	NKAIN4	
	CDHR5	
	TBX4	
	CRABP2	
	TLX1	
	ALOX12P2	
	TMEM130	
	C2orf54	
	GPR109B	
	MT1G	
	ODZ3	
	TMEM151A	
	S100A12	
	CYP4F3	
	C10orf81	
	PPP4R4	

	GCNT3	
	CDK5R2	
	HOXA7	
	SUSD4	
	CAPN8	
	HCN4	
	PRSS33	
	LOC201651	
	RANBP17	
	WNT9A	
	CCDC129	
	FRMPD1	
	GLT25D2	
	ZBTB7C	
	SLC14A2	
	C2orf65	
	AMH	
	CDH12	
	F5	
	LOC554202	
	TDGF1	
	LAMA1	
	TSPAN1	
	BHMT	
	ISM2	
	FEV	
	GJA3	
	LRRN4	

	PLAC8	
	GHRL	
	DRD1	
	DNAH2	
	DSG1	
	HGD	
	TP63	
	TAC3	
	HMP19	
	C6orf176	
	ENTPD3	
	LOC440173	
	ASCL2	
	CASP5	
	MPV17L	
	RET	
	UPK2	
	SLC5A9	
	CASKIN1	
	MIOX	
	SLC29A4	
	TPH1	
	AKR1C4	
	KLC3	
	TMEM82	
	NLRP6	
	SLCO1A2	
	ACHE	

	MME	
	FRAS1	
	ARL14	
	NCAM2	
	IDO1	
	CXCL14	
	EFNA2	
	MGAM	
	TGM1	
	NALCN	
	NAT8B	
	RTBDN	
	SEZ6L	
	MCOLN3	
	BEX5	
	WDR87	
	NEURL3	
	PALM3	
	CTSG	
	NPY6R	
	ARC	
	SHH	
	FXYD2	
	NTRK2	
	THPO	
	BCL11A	
	IL8	
	MYO3A	

	MRAP2	
	TRPM5	
	IRX5	
	SLC16A9	
	RNF183	
	MT1A	
	C3orf15	
	ATP6V1B1	
	KCNH3	
	PLD4	
	COX6B2	
	KLHDC7A	
	FMO1	
	ESYT3	
	C1orf61	
	NEBL	
	IL13RA2	
	PF4	
	GLP1R	
	C19orf69	
	IBSP	
	COL21A1	
	LOC220594	
	LY6G6C	
	MUC20	
	SLC28A3	
	HOXC11	
	TESC	

	GPD1	
	ALDH1A1	
	ALOX15	
	COL28A1	
	SLC30A10	
	DHRS9	
	ERBB4	
	TNN	
	NOL4	
	KY	
	ASCL1	
	GFI1B	
	CECR7	
	HAS1	
	SPDYC	
	AKR1E2	
	BIRC7	
	SH2D5	
	CALHM3	
	XDH	
	GLP2R	
	PLA2G10	
	HSD17B2	
	HRASLS	
	HMGA2	
	RNF126P1	
	NEUROD2	
	SLPI	

	VEPH1	
	S100A14	
	C14orf115	
	FAM55B	
	CSF2	
	C3orf67	
	CHRNA7	
	C8orf80	
	C2CD4A	
	FIGF	
	ABCG5	
	GABRE	
	C11orf41	
	HNF4A	
	AQP12B	
	GABRA4	
	HAP1	
	SCT	
	TDRD1	
	BARX2	
	TRIM72	
	HLA-G	
	AIM2	
	SRMS	
	HOXA9	
	PIRT	
	ECHDC3	
	SYCP2L	

	SMC1B	
	PDZD3	
	PNMT	
	PCDHAC1	
	GABRA2	
	AICDA	
	MYBPC2	
	WNT10A	
	NFE2	
	NPHS1	
	CNFN	
	COL29A1	
	CACNA1I	
	GPT	
	TM4SF5	
	GPR37	
	TEX19	
	RGS7	
	SLITRK3	
	MGC29506	
	FLJ16779	
	SP5	
	GDF6	
	CCL15	
	SLC22A3	
	PCA3	
	HBA2	
	PRSS3	

	CNTNAP3	
	CXCR1	
	MFAP5	
	ATP2C2	
	LOC284578	
	HSD3B2	
	SULT2B1	
	HEPHL1	
	ZNF556	
	FGF9	
	RNF212	
	FLG	
	TACSTD2	
	CXorf48	
	NXPH4	
	GPR81	
	MFSD6L	
	PCSK1	
	ONECUT2	
	ESPN	
	C17orf73	
	MAPK15	
	RHOV	
	INSC	
	DIO3	
	CHODL	
	CHST13	
	DMRTA1	

	ZNF385B	
	CLDN9	
	CCL18	
	IL1RL1	
	CST2	
	STRA6	
	POU2AF1	
	FER1L4	
	CALY	
	KCNT1	
	EGF	
	PDZK1IP1	
	CCL14-CCL15	
	LPPR1	
	TTPA	
	ZFR2	
	MUC1	
	SHD	
	EVX1	
	TCAM1P	
	SGK2	
	HAPLN1	
	KCNJ15	
	LOC284749	
	COL4A5	
	SCUBE1	
	SEC14L5	
	TFR2	

	HS3ST5	
	PRR18	
	PRLR	
	C21orf29	
	UTS2R	
	SLC6A7	
	SHISA6	
	DNAH14	
	LPPR3	
	EYA4	
	CD207	
	CXCR2P1	
	ZNF334	
	ANKS4B	
	KIAA1751	
	CYP2E1	
	LOC284233	
	CYP3A5	
	C1orf173	
	RAET1L	
	ENPP3	
	AMPD1	
	LYPD3	
	MKX	
	IL1A	
	AHNAK2	
	TNNC2	
	NPFFR2	

	NKD2	
	ABP1	
	CASR	
	KRT80	
	C4A	
	FAM150A	
	PLIN1	
	CYP4X1	
	CAMK2B	
	PROC	
	NANOS3	
	KERA	
	DLX2	
	NRG4	
	TPSG1	
	EPS8L3	
	CES3	
	RPPH1	
	FOXA2	
	COCH	
	SERPINA1	
	TTLL6	
	MYOM1	
	IL6	
	PCDH8	
	DLX6AS	
	ANKFN1	
	TREH	

	CCL24	
	LRRC15	
	FAM90A1	
	THNSL2	
	MAB21L2	
	SLC30A3	
	DLGAP1	
	C9orf169	
	GABBR2	
	UNC80	
	FLRT3	
	F13A1	
	USH1C	
	RGS6	
	FOXA1	
	CASQ1	
	MMEL1	
	LILRA4	
	CNTN3	
	C9orf152	
	NEFM	
	TPD52L1	
	KEL	
	FST	
	HES2	
	RIMS2	
	DGKB	
	HLA-DQB2	

	VAX2	
	PPARGC1A	
	C1orf161	
	AKR1C1	
	LOC100216001	
	C6orf222	
	C5orf23	
	GALNT9	
	MIA2	
	CBLN1	
	DUOX1	
	L1TD1	
	SLC38A5	
	TMPRSS5	
	PPYR1	
	PCDH11X	
	UNC45B	
	FAM66D	
	HBB	
	PCDHB8	
	XIRP1	
	F10	
	LRRC66	
	CXCL1	
	ULBP2	
	ADRA2C	
	SLC4A4	
	ZNF683	

	C11orf53	
	RHBG	
	TNFRSF13C	
	SYT9	
	SLC1A2	
	TERT	
	CILP2	
	C4orf31	
	MYPN	
	DOC2A	
	ENAM	
	TH	
	JPH1	
	PSORS1C1	
	CLCN1	
	HOXC8	
	LOC286467	
	CXCL11	
	ST6GAL2	
	FOXL2	
	DRD2	
	CXCL9	
	ZNF257	
	CACNA2D1	
	SLC44A4	
	GDF10	
	KISS1R	
	C11orf20	

	RBM11	
	GREM1	
	HAMP	
	DLX4	
	LOC441666	
	DMRT3	
	CHL1	
	HAS3	
	ZNF492	
	CELF5	
	NACA2	
	KIAA1644	
	CCL7	
	DNAH3	
	EMID2	
	LIX1	
	PODXL2	
	UGT3A2	
	LHFPL4	
	C10orf93	
	ADAMTS16	
	SPTBN4	
	LOC728819	
	SLITRK4	
	LYZ	
	EGR4	
	RORC	
	FAM180A	

	HCG22	
	MTNR1A	
	KCNA2	
	TBX18	
	IL1RL2	
	SLC24A5	
	SYCP2	
	LOC100127888	
	TREML2	
	ENDOU	
	STK33	
	PCDHB6	
	LPHN3	
	IL31RA	
	ACTA1	
	LOC728643	
	MB	
	TTLL7	
	GRB14	
	SCIN	
	ELAVL3	
	SLC7A2	
	CXCL3	
	SCUBE2	
	TMPRSS3	
	SNORD15B	
	ATP2B2	
	PTPLA	

	GPX2	
	CSTA	
	PCDHA4	
	RHBDL2	
	PAK7	
	SFTPD	
	TFCP2L1	
	LOC96610	
	AKR1C2	
	TRIM40	
	SLC13A3	
	IL33	
	CECR2	
	RAB3C	
	ACSL6	
	SLITRK2	
	ARHGEF4	
	NOXO1	
	FAM132A	
	CLIC6	
	PLAC1	
	GRIA4	
	DAB1	
	SLC35F3	
	DLX5	
	EFHC2	
	PI15	
	ARMC3	

	SDR42E1	
	P2RX2	
	PRKG2	
	GSDMC	
	MYADML2	
	FAM23A	
	FAM169B	
	QPRT	
	PMP2	
	AREG	
	DQX1	
	LOC283174	
	NUP62CL	
	ZNF541	
	CYS1	
	FGF5	
	FLJ42875	
	PCDH19	
	SPON1	
	PKD1L2	
	UBXN10	
	FGF14	
	IL1R2	
	PAPPA2	
	CCDC136	
	CNGA3	
	PRG4	
	LPAR3	

	WNT2	
	RTN4RL1	
	EMR3	
	C17orf55	
	CXCL10	
	BMP5	
	PRSS22	
	C13orf38	
	SULT1A2	
	ABCC6P1	
	ARMC4	
	AMN	
	C19orf59	
	PRSS27	
	SLCO4C1	
	LAMC3	
	IGSF9B	
	TRIM50	
	ABO	
	HAR1B	
	GRIA1	
	ALOX15B	
	ATCAY	
	RAET1G	
	PRND	
	IGSF10	
	SLC10A4	
	LGALS12	

	CABP4	
	SLIT1	
	GYLTL1B	
	GSC	
	SYNC	
	RGPD7	
	KCNG3	
	HLA-DRB5	
	MLPH	
	BTBD16	
	NWD1	
	HOXC9	
	CUX2	
	CHRM1	
	KCNQ4	
	GRIN1	
	HPGD	
	FNDC1	
	ZNF98	
	BNIPL	
	DDN	
	C3orf32	
	AMDHD1	
	PIPOX	
	EVPLL	
	GAS1	
	SIGLEC14	
	MASP1	

	PDE11A	
	SDK2	
	TNFRSF19	
	PLA2G4E	
	PDZK1	
	SMOC2	
	RAB3B	
	CRYM	
	MESP1	
	PP14571	
	NRG3	
	FIGN	
	LUZP2	
	PDE4C	
	ABCB5	
	GJB3	
	FAT2	
	NRCAM	
	MYO3B	
	LYPD6	
	APLP1	
	ISL1	
	HBA1	
	SCNN1A	
	DPF1	
	C9orf122	
	ART3	
	HS3ST2	

	ALG1L	
	KCNK9	
	MFSD4	
	LOC400696	
	ENPP5	
	ESPNL	
	FAM95B1	
	SOX8	
	SLC26A7	
	SPOCK1	
	CKM	
	COL22A1	
	PTPRR	
	KIAA1239	
	OR7D2	
	PPP1R3C	
	MAPK8IP2	
	KPNA7	
	FAM101A	
	GPR15	
	TMEM61	
	GPR120	
	VWA3B	
	LIPH	
	NTNG1	
	TEX11	
	SLN	
	LRRC26	

	NSUN7	
	NAT8	
	SEZ6	
	IGFN1	
	CA12	
	CYP21A2	
	LOC100131726	
	HNF1B	
	DNAH8	
	CMBL	
	CPXM2	
	BCL2L10	
	HLA-DOB	
	CASC1	
	PLCH2	
	CCL23	
	LOC647946	
	LOC100128164	
	ADAM22	
	ZNF883	
	GPR133	
	CRIP3	
	VGF	
	FOSB	
	ADAMTS18	
	AGXT	
	MTUS2	
	TAF7L	

	CXCR2	
	KRT1	
	FOXN1	
	FAM198A	
	TNFRSF11B	
	MYLK	
	RAP1GAP	
	NCF1B	
	GRM4	
	KLHL35	
	LGI3	
	LOC154822	
	KCNK10	
	DNAH5	
	GPR1	
	C20orf103	
	CDH22	
	LRRC4C	
	TNNT2	
	TRPV3	
	C17orf93	
	RDH12	
	CLDN11	
	HPD	
	SLC27A6	
	DBC1	
	BCL2L15	
	LOC286002	

	NDP	
	SUCNR1	
	PLEKHB1	
	SVOP	
	EPHX4	
	CDH2	
	PEG3	
	LRP4	
	OVOL1	
	IL17A	
	TG	
	FAM153A	
	ASPA	
	ANKRD20A3	
	LY9	
	UPB1	
	WIPF3	
	MMP11	
	DPEP3	
	MAOB	
	BTN1A1	
	HLF	
	CHI3L1	
	CHST8	
	ENTPD2	
	HAPLN4	
	ULBP1	
	ACSM3	

	IP6K3	
	SPP1	
	FCRL6	
	DEPDC7	
	TPSB2	
	CNGB1	
	FAM46B	
	CPA3	
	TRIM7	
	NPM2	
	CCL11	
	GZMK	
	NAV3	
	HTR2A	
	FAM5C	
	ADAMTS15	
	C5orf49	
	SPESP1	
	RYR3	
	DIRAS1	
	ELAVL4	
	GALNT5	
	DGCR5	
	DUSP15	
	GLYATL1	
	PRSS30P	
	KCNJ12	
	C14orf64	

	LOC100133545	
	SH3GL2	
	HCG4	
	CCL13	
	WDR66	
	DLK2	
	C6orf155	
	SCML2	
	C19orf33	
	TMEFF2	
	CDKL2	
	RIBC2	
	KCNA5	
	C21orf88	
	INA	
	PDGFRL	
	MGP	
	GUCA1A	
	KCNMB1	
	BAMBI	
	CD1A	
	NOG	
	TNFRSF6B	
	C2orf70	
	PTCH2	
	VWC2	
	IKZF3	
	KIRREL3	

	LOC100130238	
	DACH1	
	ITIH4	
	STX19	
	POU2F3	
	SORBS1	
	UNC5CL	
	EPN3	
	STAC	
	KISS1	
	LOC162632	
	ROBO2	
	C1QL4	
	TDGF3	
	ZNF835	
	KLHL13	
	PROK2	
	KCNJ5	
	PPP1R1C	
	FAM135B	
	CYP2D6	
	NEK5	
	ZSCAN23	
	NOX5	
	IL1RN	
	SYT3	
	C12orf27	
	IFNG	

	ITGB6	
	GUCY1B2	
	KIAA1199	
	LOC284551	
	GOLT1A	
	FAM163A	
	ZPLD1	
	PPFIA2	
	OTOF	
	PANX2	
	FAM84A	
	FGF10	
	FAM43B	
	KIAA0319	
	GPC5	
	CACNA2D3	
	SIX1	
	MYCN	
	BMX	
	CCL28	
	PRRT4	
	NPAS4	
	UBD	
	PLA2G4D	
	CCDC80	
	C1orf186	
	FZD10	
	LOC401387	

	C3orf32	
	AMDHD1	
	PIPOX	
	EVPLL	
	GAS1	
	SIGLEC14	
	MASP1	
	PDE11A	
	SDK2	
	TNFRSF19	
	PLA2G4E	
	PDZK1	
	SMOC2	
	RAB3B	
	CRYM	
	MESP1	
	PP14571	
	NRG3	
	FIGN	
	LUZP2	
	PDE4C	
	ABCB5	
	GJB3	
	FAT2	
	NRCAM	
	MYO3B	
	LYPD6	
	APLP1	

	ISL1	
	HBA1	
	SCNN1A	
	DPF1	
	C9orf122	
	ART3	
	HS3ST2	
	ALG1L	
	KCNK9	
	MFSD4	
	LOC400696	
	ENPP5	
	ESPNL	
	FAM95B1	
	SOX8	
	SLC26A7	
	SPOCK1	
	CKM	
	COL22A1	
	PTPRR	
	KIAA1239	
	OR7D2	
	PPP1R3C	
	MAPK8IP2	
	KPNA7	
	FAM101A	
	GPR15	
	TMEM61	

	GPR120	
	VWA3B	
	LIPH	
	NTNG1	
	TEX11	
	SLN	
	LRRC26	
	NSUN7	
	NAT8	
	SEZ6	
	IGFN1	
	CA12	
	CYP21A2	
	LOC100131726	
	HNF1B	
	DNAH8	
	CMBL	
	CPXM2	
	BCL2L10	
	HLA-DOB	
	CASC1	
	PLCH2	
	CCL23	
	LOC647946	
	LOC100128164	
	ADAM22	
	ZNF883	
	GPR133	

	CRIP3	
	VGF	
	FOSB	
	ADAMTS18	
	AGXT	
	MTUS2	
	TAF7L	
	CXCR2	
	KRT1	
	FOXN1	
	FAM198A	
	TNFRSF11B	
	MYLK	
	RAP1GAP	
	NCF1B	
	GRM4	
	KLHL35	
	LGI3	
	LOC154822	
	KCNK10	
	DNAH5	
	GPR1	
	C20orf103	
	CDH22	
	LRRC4C	
	TNNT2	
	TRPV3	
	C17orf93	

	RDH12	
	CLDN11	
	HPD	
	SLC27A6	
	DBC1	
	BCL2L15	
	LOC286002	
	NDP	
	SUCNR1	
	PLEKHB1	
	SVOP	
	EPHX4	
	CDH2	
	PEG3	
	LRP4	
	OVOL1	
	IL17A	
	TG	
	FAM153A	
	ASPA	
	ANKRD20A3	
	LY9	
	UPB1	
	WIPF3	
	MMP11	
	DPEP3	
	MAOB	
	BTN1A1	

	HLF	
	CHI3L1	
	CHST8	
	ENTPD2	
	HAPLN4	
	ULBP1	
	ACSM3	
	IP6K3	
	SPP1	
	FCRL6	
	DEPDC7	
	TPSB2	
	CNGB1	
	FAM46B	
	CPA3	
	TRIM7	
	NPM2	
	CCL11	
	GZMK	
	NAV3	
	HTR2A	
	FAM5C	
	ADAMTS15	
	C5orf49	
	SPESP1	
	RYR3	
	DIRAS1	
	ELAVL4	

	GALNT5	
	DGCR5	
	DUSP15	
	GLYATL1	
	PRSS30P	
	KCNJ12	
	C14orf64	
	LOC100133545	
	SH3GL2	
	HCG4	
	CCL13	
	WDR66	
	DLK2	
	C6orf155	
	SCML2	
	C19orf33	
	TMEFF2	
	CDKL2	
	RIBC2	
	KCNA5	
	C21orf88	
	INA	
	PDGFRL	
	MGP	
	GUCA1A	
	KCNMB1	
	BAMBI	
	CD1A	

	NOG	
	TNFRSF6B	
	C2orf70	
	PTCH2	
	VWC2	
	IKZF3	
	KIRREL3	
	LOC100130238	
	DACH1	
	ITIH4	
	STX19	
	POU2F3	
	SORBS1	
	UNC5CL	
	EPN3	
	STAC	
	KISS1	
	LOC162632	
	ROBO2	
	C1QL4	
	TDGF3	
	ZNF835	
	KLHL13	
	PROK2	
	KCNJ5	
	PPP1R1C	
	FAM135B	
	CYP2D6	

	NEK5	
	ZSCAN23	
	NOX5	
	IL1RN	
	SYT3	
	C12orf27	
	IFNG	
	ITGB6	
	GUCY1B2	
	KIAA1199	
	LOC284551	
	GOLT1A	
	FAM163A	
	ZPLD1	
	PPFIA2	
	OTOF	
	PANX2	
	FAM84A	
	FGF10	
	FAM43B	
	KIAA0319	
	GPC5	
	CACNA2D3	
	SIX1	
	MYCN	
	BMX	
	CCL28	
	PRRT4	

	NPAS4	
	UBD	
	PLA2G4D	
	CCDC80	
	C1orf186	
	FZD10	
	LOC401387	
